# Decoding the multifaceted role of erythrocyte PMCA4b in oxidative stress-mediated malaria protection and artemisinin resistance

**DOI:** 10.1128/mbio.01738-25

**Published:** 2025-12-16

**Authors:** Priya Agrohi, Swati Garg, Shreeja Biswas, Preeti Maurya, Vijay Kumar, Jyoti Sharma, Bidhan Goswami, Samir Kumar Sil, Mrigendra Pal Singh, Sunil Kumar Chand, Sneh Shalini, Om P. Singh, Gunanidhi Dhangadamajhi, Neelima Mishra, Sivaprakash Ramalingam, Prashant Kumar Mallick, Shailja Singh

**Affiliations:** 1 ICMR-National Institute of Malaria Research 28861, New Delhi, India; 2 Academy of Scientific and Innovative Research (AcSIR) 550336, Ghaziabad, Uttar Pradesh, India; 3 Special Centre for Molecular Medicine, Jawaharlal Nehru University 28754, New Delhi, India; 4 Department of Life Sciences, Sharda University 193167, Greater Noida, Uttar Pradesh, India; 5 Department of Human Physiology, Molecular Genetics and Cancer Biology Laboratory, Tripura University 72896, Suryamani Nagar, Tripura, India; 6 Department of Human Physiology, Tripura University 72896, Suryamaninagar, Tripura, India; 7 ICMR-National Institute of Malaria Research Field Unit Jabalpur, Jabalpur, Madhya Pradesh, India; 8 Indian Council of Medical Research 28604, New Delhi, India; 9 Department of Biotechnology, North Orissa University 78111 , Baripada, Odisha, India; 10 CSIR-Institute of Genomics and Integrative Biology (CSIR-IGIB) 28840, New Delhi, India; The George Washington University Milken Institute of Public Health, Washington, DC, USA

**Keywords:** malaria, artemisinin resistance, PMCA4b, ATP2B4, oxidative stress, resveratrol

## Abstract

The ATP2B4 gene, encoding the PMCA4b Ca²^+^-ATPase in erythrocytes, has been linked to malaria protection via genome-wide association studies, though the proposed dehydration mechanism remains unclear. This study evaluates ATP2B4 genotypes and PMCA4b expression in malaria susceptibility and artemisinin sensitivity. ATP2B4 genotypes were compared across severe malaria, uncomplicated malaria, and healthy controls in Indian population. PMCA4b expression, intra-erythrocytic calcium, oxidative stress markers, Gardos channel activity, and *Plasmodium falciparum* growth dynamics were analyzed. Artemisinin sensitivity was assessed using growth inhibition and ring survival assays. ATP2B4 genotypes showed no significant association with malaria protection. Regardless of genotype, low PMCA4b expression correlated with increased intra-erythrocytic calcium, oxidative stress, and reduced *in-vitro* parasite growth. Gardos channel activity inversely correlated with PMCA4b but did not induce dehydration. Instead, oxidative-stress regulation by PMCA4b emerged as a key factor in malaria protection. Treatment of RBCs with resveratrol, a PMCA inhibitor, further validates the functional role of PMCA in regulation of intracellular calcium and oxidative stress. Notably, low PMCA4b expression also reduced *P. falciparum* artemisinin sensitivity, providing evidence that host genetic variation can influence drug efficacy. These findings suggest a role for PMCA4b in oxidative stress and drug response as critical to malaria pathophysiology. We further emphasize that many redox modulating RBC polymorphisms in malaria endemic areas could also influence artemisinin efficacy and serve as potential biomarkers for predicting therapeutic response.

**IMPORTANCE:**

Discovery of the mechanism by which human host variations affect the sensitivity of artemisinin in parasite provides an interestingly important view for optimizing anti-malarial treatment strategies. The human host and malaria parasite share a closely interconnected relationship; hence, we propose that parasite physiology or drug resistance cannot be studied alone without considering the host’s biological factors. This study demonstrates that increase in intracellular calcium of RBCs is associated with a proportionate increase in intracellular oxidative stress, which affects the artemisinin sensitivity. Thus, variations in PMCA4b expression, the primary calcium efflux pump in RBCs, significantly alter the erythrocytic ROS levels, thereby affecting the *P. falciparum* growth and artemisinin sensitivity. Notably, intracellular redox imbalance is a common phenotype of multiple erythrocytic polymorphisms prevalent in malaria endemic areas, such as sickle cell, thalassemia, G6PD deficiency, etc. This study advocates the need for a widespread population-based investigation that associates the importance of host erythrocyte oxidative microenvironment surveillance in monitoring antimalarial drug resistance.

## INTRODUCTION

Malaria elimination requires the concerted efforts of all nations to achieve the goal of a world free of malaria. However, the disease remains the major cause of child mortality worldwide, killing more than a million children in Africa alone each year even after introduction of various antimalarial therapies (1, 2). World Health Organization (WHO) has recently put forward a global technical strategy for malaria elimination by 2030 and recommends the stronger surveillance of antimalarial drug efficacy and resistance.

Malaria has co-evolved with the human host for ages and has acted as one of the strongest selection forces on the human genome (2). It is suggested that many human genetic polymorphisms are the result of selection for malaria resistance. For example, malaria-endemic regions have witnessed the evolutionary selection of blood group antigens, hemoglobin chains, and human lymphocyte antigen (HLA) classes that are protective of malaria (3, 4). Several erythrocyte defects that provide protection against malaria, such as sickle-cell disease, thalassemia, glucose-6-phosphatase deficiency, etc., are prevalent in endemic areas (5). In addition, introduction of antimalarial drugs due to human intervention is altering parasite population dynamics, resulting in the emergence of multi-drug-resistant genotypes (6). Presumably, when humans were evolving genetically to fight malaria, the parasite was evolving to become resilient toward drug interventions; although these evolutionary events did not occur simultaneously, both reflect parallel pressures for survival.

Until now artemisinin-based therapies have been the backbone of malaria treatment, due to their effectiveness against *Plasmodium falciparum* malaria. However, recent reports of artemisinin resistance (ART-R) have raised concerns about the sustainability of these therapies. ART-R is characterized by reduced killing of ring-stage *P. falciparum*, resulting in delayed parasite clearance (7). ART-R has been linked with the mutations in the Pfkelch13 protein propeller domain (8); however, there are reports of artemisinin treatment failure without any mutations in K13 protein or other genes of the parasite (9, 10). Other factors, such as sequestration of parasites to distant locations, further reduce the efficacy of artemisinin and delay parasite clearance (11). Recently host erythrocyte redox oxidative stress (ROS) had also been linked with resistance of malaria parasite toward artemisinin (12). This report indicated that in RBCs, oxidative stress induced *in vitro* can influence the sensitivity of antimalarial drug. Interestingly, an earlier study demonstrated that *P. falciparum* infecting alpha-thalassemic RBCs shows reduced sensitivity to artemisinin, largely due to altered drug accumulation capacity of variant RBCs (13). However, the effect of human host variations that modulate the RBC oxidative stress on artemisinin resistance has not been investigated yet. Even a very recent study indicated the role of two other parasite encoded proteins, PfRAD5 and PfWD11, in ART-R but still misses out on the contribution of the host in this phenomenon (14, 15).

Intraerythrocytic oxidative stress can be linked with many physiological factors, including the intracellular calcium levels of RBCs (16–18), which are primarily regulated by the plasma membrane calcium ATPase (PMCA4b). PMCA4b is encoded by ATP2B4 gene, which has emerged as a strong malaria resistance candidate in several GWAS and case-control studies in various parts of the world. Variation in the regulatory region of ATP2B4 was found to have a very strong association with resistance to severe *P. falciparum* malaria in recent studies conducted in Kenya, Ghana, and across many sites in Africa, Asia, and Oceania (19–26). These variations present in the ATP2B4 gene decreased the likelihood of SM (severe malaria) by 40% in African children while also reducing the odds of placental *P. falciparum* malaria in African women by 64% (27). These independent and diverse research findings strongly indicate the significant role of ATP2B4 gene in protection toward infection of malaria parasites to human host and suggest RBC dehydration as the causal mechanism (28) (Table S1). Despite various research, the experimental basis of this remains uncleared. Although linked to protection in human studies, a murine model showed that PMCA4 (ATP2B4) does not affect parasite levels but contributes to experimental cerebral malaria (29). Furthermore, the role of PMCA4b-mediated regulation of intracellular calcium in redox imbalance and antimalarial drug resistance has not been investigated yet.

This study comprehensively investigated the relationship of human genetic variations in ATP2B4 gene with the resultant surface expression of PMCA4b, the intraerythrocytic levels of calcium, calcium-dependent potassium channel (gardos channel) activation, reactive oxygen species (ROS), *in-vitro* parasite growth, and the resistance toward artemisinin in the intraerythrocytic parasite. We demonstrate that the protein expression of PMCA4b negatively correlates with the intraerythrocytic calcium, Gardos channel activity, and ROS levels but does not show any association with RBC hydration. Furthermore, the *in-vitro* parasite growth is also reduced in RBCs with low PMCA4b expression, and this study suggests that oxidative stress is the main factor for malaria susceptibility rather than variations in RBC dehydration. Further *in-vitro* growth inhibition assays and ring survival assays results revealed that artemisinin efficacy is significantly reduced in the parasites growing within the RBCs having low PMCA4b (high calcium and ROS). This study unravels the role of host PMCA4b in conferring malaria susceptibility and artemisinin resistance to the parasite. Several studies reported artemisinin resistance in malaria endemic areas without finding any genetic evidence for this occurrence. Our data provide an explanation for this and advocate the need to consider the human factors while assessing this phenomenon. Thus, there is need for population-specific research to understand the role of host genetic factors influencing artemisinin resistance in the malaria parasite.

## MATERIALS AND METHODS

### Study population and sample collection

A total of 235 samples were used in this study; 117 blood samples were collected from healthy volunteers at NIMR Delhi in the form of dried blood spot (DBS) by finger pricking for DNA isolation. DNA samples from febrile malaria negative (*n* = 40) (manuscript in preparation), uncomplicated *P. falciparum* malaria (*n* = 54) (manuscript in preparation), and severe malaria cases (*n* = 24) (30) were taken from other studies. For other experiments, 2 mL venous blood was collected from malaria-negative apparently healthy donors after getting informed consents. Exclusion criteria for this study involve G6PD deficiency, sickle, and thalassemia hemoglobinopathies. The information of all the samples used in this study is available in Table S5.

### Selection of SNPs and annotation of selected polymorphism

Many SNPs associated with malaria resistance have been identified in previous studies. Although these SNPs are predominantly located in regulatory and non-coding regions spanning the first to third exons of the gene, this study prioritizes those with robust GWAS signals or replicated results from experimental studies (Table S1). UCSC Genome Browser (31) was used to analyze their functional significance and potential regulatory roles.

### DNA isolation, PCR amplification, and genotyping

Genomic DNA isolation was performed on dried blood spots (DBS) using the QiaAmp DNA minikit following the manufacturer’s instructions (Qiagen, Hilden, Germany). Three sets of primers were designed using Primer-BLAST software to amplify the 3 kb regulatory DNA sequence of the ATP2B4 gene, including the enhancer region, 5' untranslated region (5'UTR), and second intronic region. Initial PCR amplification involved a 3 kb DNA fragment using forward primers for the enhancer region and reverse primers for the 2nd intron. A nested PCR was subsequently performed to amplify all three regions (Table S4). Nested PCR of 20 µL reaction volume used 1 µL of template DNA from the primary PCR. Some samples displayed variability in the amplification of the enhancer region, which required adjustments in primer sets (F 5’−1509-AAAAGGCTGATGTGTGGCAG-3′, R 5′-CGGCACCAAGTTTTGTTCTGA-3′). PCR products were electrophoresed on a 2% agarose gel (Promega/Ameresco) in 0.5 TBE buffer and visualized under UV transillumination at 302 nm using a gel documentation system. The PCR products (887 bp, 749 bp, and 603 bp) were cleaned using ExoSAP-IT (Thermo Fisher Scientific) and sequenced with forward and reverse primers using ABI BigDye Terminator v3.2 (Thermo Fisher Scientific). The sequences were aligned and compared with the reference human genome GRCh38 (Genome Reference Consortium Human Reference 38) and analyzed for nucleotide variations using Mega 11.0 software (32), followed by evaluations of genotype and allele frequency across different groups.

The distribution of genotypes (major, minor, and heterozygous) for each regulatory region (Enhancer, 5’UTR, and 2nd intron) across the four malaria outcome groups was analyzed using χ^2^ test for independence. The test assessed whether the distribution of genotypes was independent of the malaria outcome groups or if there were significant differences in genotype prevalence across groups. All analyses were performed with a 95% CI, and *P*-value less than 0.05 was considered statistically significant.

### HWE, sequence phasing, and linkage disequilibrium

Hardy-Weinberg equilibrium (HWE) analysis was performed based on allele frequencies to assess whether all alleles were in equilibrium. For linkage disequilibrium analysis, DNA sequence data were phased using DNAsp software (version 6) (33). Phasing was performed for accurately inferring the haplotype phase of the diploid sequences, essential for proper estimation of haplotype diversity and other population genetic parameters. The phasing process utilized algorithms from HAPAR, PHASE , and fast PHASE (34–36). The level of linkage disequilibrium (LD) within the samples was measured using R² and D' statistics, and the total number of haplotypes and segregating sites was determined using DNAsp software. Pairwise linkage disequilibrium for each genetic variation was computed and LD plots were generated using TASSEL (37) and GraphPad Prism 8.0.1.

### Screening for hemoglobinopathies and G6PD deficiency

To eliminate potential confounding factors due to underlying hematological abnormalities, all donor samples were screened for G6PD deficiency, hemoglobinopathies, and general hematological health. G6PD enzyme activity was measured using the STANDARD G6PD Analyzer (SD Biosensor), a portable, quantitative point-of-care device. For hemoglobinopathy screening, the Sickle SCAN rapid diagnostic test (BioMedomics) was used. This lateral flow immunoassay detects the presence of hemoglobin A, S, and C, allowing identification of individuals with sickle cell trait (HbAS), sickle cell disease (HbSS), and HbC variants. Only donors with normal hemoglobin (HbAA) and normal G6PD activity were included in the study. In addition, complete blood counts (CBC) were performed for all donors using an automated hematology analyzer to ensure that hematological parameters fell within normal reference ranges. Donors with any abnormal findings were excluded from further analyses.

### PMCA4b protein expression and localization on RBCs

PMCA4b protein expression was measured by flow cytometry-based assay. In brief, 10–20 µL washed RBCs from each donor with different ATP2B4 genotypes was fixed with 2.5% glutaraldehyde/PBS for 30 min at 4°C, permeabilized with 0.01% Triton X100/PBS for 10 min at room temperature (RT), and blocked with 3% BSA in PBS. Permeabilized RBCs were then incubated with Anti-PMCA4b clone JA3 primary antibody (Merck Millipore) in 1%BSA/PBS for 1 h at RT followed by secondary antibody, Alexa Fluor 488 goat labeled anti-mouse antibody (Life Technologies), in 1% BSA in PBS for 1 h at RT. After antibody labeling, thin blood smear was made for the localization study, and the same samples were acquired using AMNIS imaging flow cytometer (Cytek AMNIS FlowSight, Manufacturer: Luminex Corporation, Austin, TX, USA). The results were analyzed using AMNIS IDEAS software, version 6.4, to determine MFI. Based on the imaging data, single cell population was selected excluding the doublets, clumps, debris, and mean fluorescence intensity was measured. Thin blood smear slide was examined using a fluorescence microscope (Olympus, Shinjuku, Tokyo, Japan) with 100× objective for localization study. To validate the specificity of the anti-PMCA4b antibody, ghost membranes were prepared from washed donor RBCs by hemolysis in hypotonic lysis buffer (0.1× PBS; Gibco) and subsequently washed until most of the hemoglobin was removed and a white pellet was obtained (38), whereas parasite lysates were obtained from late-stage *P. falciparum* infected RBCs following selective saponin lysis (0.05% wt/vol; Sigma-Aldrich) and thorough PBS washes to eliminate any residual component from RBCs (39). Equal amounts of protein from both lysates (60 µg per lane) were resolved by SDS-PAGE and transferred to a nitrocellulose membrane (0.2 µm; Bio-Rad) for western blotting using anti-PMCA4b monoclonal antibody (clone JA3; Merck Millipore) followed by HRP-conjugated goat anti-mouse secondary antibody (Invitrogen).

### Intracellular basal calcium concentration and calcium efflux assay

Intracellular calcium concentration was assessed in the RBCs from different ATP2B4 genotypes and varying PMCA4b expression with the help of Fluo-4AM calcium dye assay (40) with some modification. In brief, washed RBCs from each donor were stained with 1 µM Fluo-4-AM dye (Molecular Probes/Invitrogen) for 30 min at 37°C in dark. After incubation, dye loaded RBCs were washed with 1× PBS and analyzed with imaging flow cytometry to assess the intracellular basal calcium concentration. To measure the activity of PMCA4b, Fluo-4AM stained RBCs were loaded with calcium using 10 µM ionomycin ionophore (Molecular Probes/Invitrogen cat. 12422) in incomplete RPMI medium containing 0.42 mM calcium. Calcium efflux from the RBCs was measured by flow cytometry following PBS wash at 0 min and 15 min (41–43). Effect of resveratrol (PMCA inhibitor) on calcium efflux was also assessed by treatment with 50 µM resveratrol.

#### Control experiments

Control experiments (*N* = 6) were performed to assess Fluo 4AM dye retention in RBCs (44). Briefly, RBCs were loaded with 1 µM Fluo-4-AM (30 min, 37°C, dark) in the presence and absence of probenecid (2.7 mM). After incubation, samples were washed with PBS and fluorescence was acquired by flow cytometry at 3 h and 6 h time points. To evaluate complete ionomycin removal from RBC membrane, BSA + PBS washing was compared with PBS washing (45). A separate control experiment tested resveratrol treatment (50 µM, 2 hr) after either PBS or BSA washing following ionomycin exposure was also performed; however, extensive PBS washing produced comparable results and was used consistently. All control experiments were analyzed by flow cytometry.

### Measurement of intraerythrocytic ROS levels

Intracellular reactive oxygen species (ROS) of RBCs was measured by DCFH-DA (2′,7′-dichlorodihydrofluorescein diacetate) (D6883, Sigma-Aldrich) oxidation-sensitive fluorescent dye, by flow cytometry. DCFH-DA is a non-fluorescence probe that can diffuse inside the RBCs where it is deacetylated by esterase, producing DCF (dichlorofluorescein), which emits fluorescence when oxidized by ROS. Washed RBCs were loaded with 10 µM DCFDA dye and incubated in the dark for 40 min; after incubation, RBCs were washed with 1× PBS and acquired by flow cytometry. Effect of PMCA inhibition or increased calcium concentration inside RBCs on ROS production was measured by treating RBCs with 50 µM resveratrol for 1 h before staining with DCFH-DA dye. For the ROS assay, control experiments were similarly performed with probenecid to check DCFDA dye retention (46) inside the RBCs.

### Erythrocytic flux OR potassium ion channel Gardos activity assay

The activity of Gardos channel in the RBCs with different PMCA4b expression was measured by a thallium-based FluxOR Potassium Ion Channel Assay (Molecular Probes, Life Technology). In brief, the iRPMI media were removed from the washed RBCs, and then, the RBCs were incubated in Flux OR II reagent dye in loading buffer (contains FluxOR reagent, a thallium-sensitive fluorogenic indicator dye) for 1 hour in dark. This was followed by washing and 30 min of exposure to assay buffer (contains probenecid [2.7 mM], an anion pump blocker that helps in retaining the thallium sensitive form of FluxOR reagent in the cytosol). This experiment was performed in 2 set of samples from which one set was treated with resveratrol (50 µM) for 2 h before the start of the experiment. The baseline fluorescence intensity was then measured for 2 min. After measuring the baseline intensity, stimulus buffer (contains thallium) was injected in each sample followed by immediate measurement of fluorescence kinetics for 4 min. The fluorescence intensity was measured at excitation 480 nm and emission 520 nm using a microplate reader.

### *P. falciparum* growth assay and artemisinin growth inhibition assay (GIA)

*P. falciparum* laboratory strain 3D7, cultured in RPMI 1640 (Invitrogen, USA), was used for most of the *in vitro* assays. *P. falciparum* was cultured in human O^+^ erythrocytes at 4%–5% hematocrit in RPMI 1640 medium supplemented with HEPES, sodium bicarbonate, Albumax II, hypoxanthine, and gentamicin, under a gas mixture of 5% CO₂, 5% O₂, and 90% N₂ at 37°C, following the standard Trager and Jensen method (47). Schizont-stage infected RBCs were purified using percoll mediated density gradient for all assays. *P. falciparum* growth assay was carried out as previously described (48). In brief, synchronized schizont-stage parasites were added to the RBCs from different ATP2B4 genotype donors, at 0.2% parasitemia and 2% hematocrit, plated in duplicate and incubated at 37°C with 5% CO2, 5% O2, and 90% N. Initial parasitemia at 0 h and final parasitemia after 48 h were measured using a flow cytometer as described previously (49) and were also validated by microscopy. Growth rates of parasites were determined using formula: (final parasitemia — initial parasitemia)/initial parasitemia. *P. falciparum* susceptibility to artemisinin in RBCs of different PMCA4b levels was assessed (50) in the presence of a range of concentrations of artemisinin from 50 nM to 1.5 nM. IC50 was calculated by plotting percent inhibition of growth against the drug concentration on GraphPad Prism version 8.0.1.

### Growth inhibition assay by PMCA4b inhibitors resveratrol and ATA

For the growth inhibition assay, PMCA inhibitors resveratrol and aurintricarboxylic acid (ATA) were used. In brief, ring stage *P. falciparum* were seeded in a 96-well plate. Both the drugs were serially diluted and plated in duplicate. Parasitemia was monitored after 72 h of incubation by counting the percentage of parasites in Giemsa-stained smears under light microscope. All experiments were performed in duplicate, and 50% inhibitory concentration (IC50) was determined using the dose-response curve generated in GraphPad Prism software.

### Erythroid progenitor BEL-A cell culture

Bel-A cells were maintained as described previously (51). Briefly, the cells were cultured in expansion medium (StemSpan SFEM II [Stemcell Technologies Inc.], 50  ng/mL SCF [Immunotools, Germany], 3 U/mL EPO [Zydus, India], and 10^−6^ M dexamethasone [Sigma-Aldrich]). The cells were maintained in this medium at a density of 1–3 × 10^5^ cells/mL at 37°C, 5% CO2. The culture medium was changed 2–3 times per week and replaced with fresh medium.

### Transfection of siRNA targeting PMCA4b encoding gene ATP2B4 in BEL-A cells

ATP2B4 knockdown was done in pro-erythroblast stage of BEL-A cells, transfected with siRNA designed against ATP2B4 at two different concentrations (150 nM and 200 nM). In control, an equal number of cells (0.5 × 10^6^) was transfected with scrambled siRNA at 1,100V, 30 ms, 3 pulse using a Neon electroporation system (Invitrogen). After 6 h of transfection, the cells were washed with 1× PBS and re-suspended in expansion medium. After 72 h of transfection, the cells were analyzed for ATP2B4 expression.

### Erythrocytic topographical analysis by atomic force microscopy

To examine the morphological changes in the RBCs from donors with high and low PMCA4b expression, RBCs were thin smeared and air-dried on a clean glass slide. Imaging was done using the WITec alpha system, using NSG30 probes with a force constant of 22–100 N/m, a resonant frequency of 240–440 Hz, and a tip curvature radius of 10 nm in non-contact mode. Topographic images were obtained at a resolution of 512 × 512 points per line with a scan rate of 0.5 times/line (Trace) (s). All the AFM images were captured using the Control Four 4.1 software and analyzed using software Project Four 4.1 software (WITec, Germany). To check the effect of resveratrol on RBC morphology, RBCs were treated with resveratrol (50 µM) for 2 h and then subjected to AFM imaging.

### Ring survival assay of 3D7 in RBCs with varying expressions of PMCA4b

*P. falciparum* laboratory strain 3D7 was cultured until parasitemia was 1%–2%. Percoll density gradient was done to synchronize the parasite culture, and schizont stage of parasite was obtained. The culture was incubated for 3 h with RBCs, with varying surface expressions of PMCA4b. Following incubation, early ring stages were exposed to 700 nM DHA for 6 h. After 6 h, the culture was centrifuged and washed thrice with RPMI incomplete medium to remove the DHA. Culture was then transfered to a 96-well plate and incubated for 66 h. This experiment was performed in duplicate and for parasitemia estimation. Giemsa-stained blood smear was prepared at each stage. The percentage of ring survival was calculated as parasitemia at 72 h in DHA-treated samples divided by the parasitemia in untreated sample processed in parallel.

### Statistical and data analyses

To check the association of genotypes (major, minor, and heterozygous) of each regulatory region (Enhancer, 5′UTR, and 2nd intron) among the four malaria outcome groups, χ^2^ test for independence was used. For all the flow cytometry experiments, 20,000 events were captured, and the results were analyzed using AMNIS IDEAS software, version 6.4, to determine MFI cells following the gated selection of singlet population.

Flow cytometer data are presented as graphs generated by GraphPad Prism 8.0.1. During the experiments, two control samples were included in every batch as a baseline, with their MFI values normalized to 1. The MFI of other samples was calculated relative to these controls. This normalization approach was necessary to avoid inter-batch variations in fluorescence measurements. For each data set, test of normality (Shapiro-Wilk test and Kolmogorov-Smirnov test) was performed to check the normal distribution; if data did not meet the assumption of normality, then Kruskal-Wallis test was performed with Dunn’s post-hoc test to compare difference between multiple groups and Spearman rho for assessing the relationship between variables. An independent *t*-test was used to compare the mean between the two groups, and one-way ANOVA was used for comparison involving more than two groups. Differences in the PMCA4b expression, intracellular calcium concentration, and intracellular ROS, across different genotypes, were evaluated using the Kruskal-Wallis test, followed by post-hoc comparisons (Dunn’s multiple comparisons test) to compare pairwise difference between groups by excluding one genotype (minor), as it has only one data point. Linear regression, Pearson correlation, and Spearman rho were used for all the correlation data. In all statistical analyses, a 95% CI was applied to ensure the reliability of the results. Data were considered statistically significant if *P* < 0.05. *P* < 0.05 represents *, *P* < 0.01 represents **, and *P* < 0.001 represents *** significance. All analyses were performed using GraphPad Prism version 8.0.1.

## RESULTS

### The complex nature of the PMCA4b encoding regulatory ATP2B4 genetic variations was not found to be associated with malaria severity in the studied Indian population

**Fig 1:**
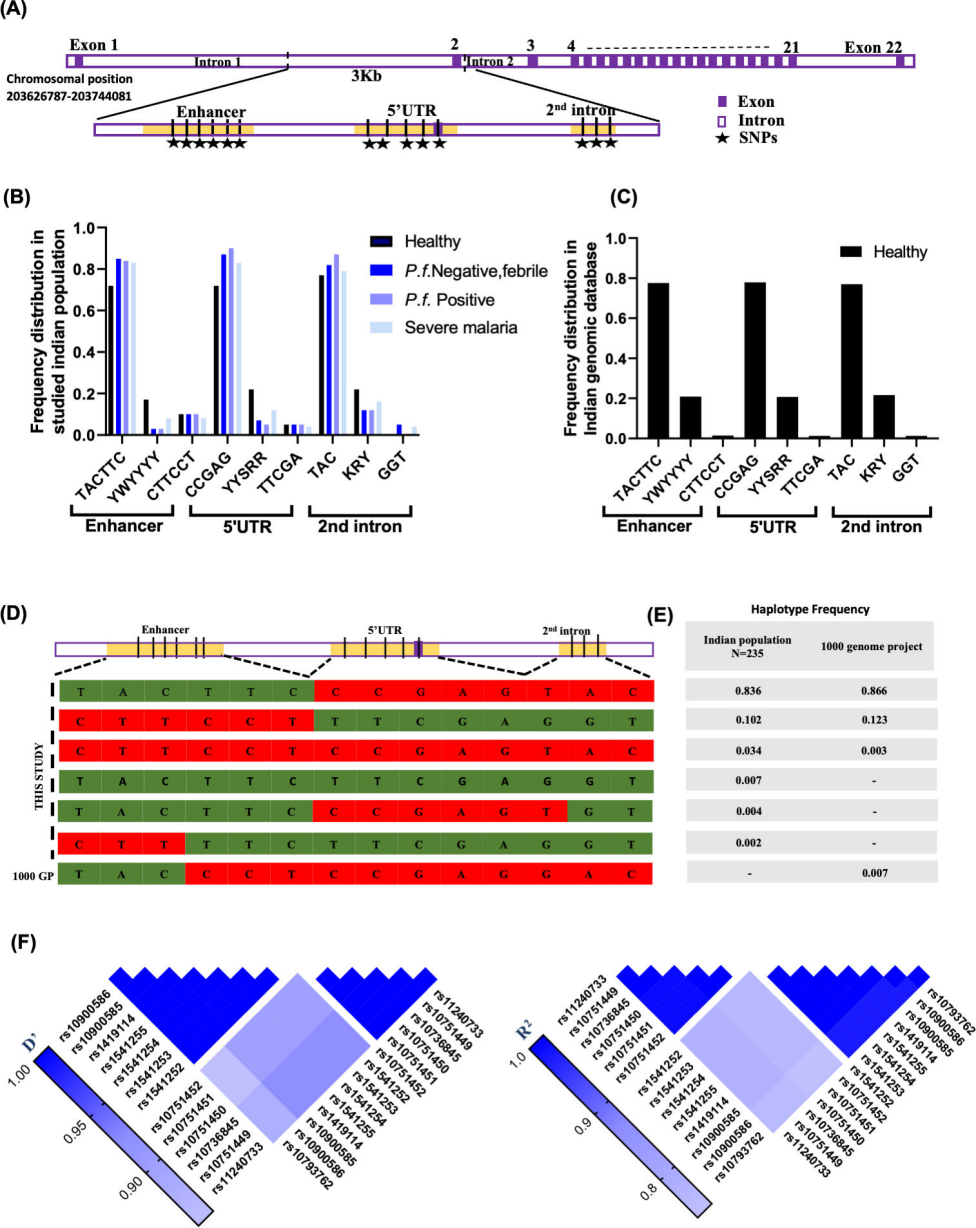
Prevalence and linkage pattern of regulatory variants of ATP2B4 gene in Indian population. (**A**) Schematic diagram of the ATP2B4 gene highlighting the three regulatory regions containing variations. A total of 14 variations were identified by Sanger sequencing (marked by star). The zoomed image illustrates the specific locations of these mutations within the regulatory regions. (**B**) Bar graph showing frequency distribution of genotypes present in enhancer, 5’UTR and 2nd intron region of the ATP2B4 gene in healthy individual (*N* = 117), Febrile malaria negative (*N* = 40), uncomplicated *P. falciparum* positive (*N* = 54), and severe malaria samples (*N* = 24). (**C**) Bar graph showing frequency distribution of the genotypes in healthy individual from IndiGenome database (*N* = 1024), showing almost the same frequency as in studied sample groups. (**D**) Distribution of haplotypes in the ATP2B4 gene among the studied Indian population, and the 1000 Genomes Project. Green and red colors represent the previously known protective and susceptible alleles. (**E**) Frequency of identified haplotypes within the studied population compared to global populations included in the 1000 Genomes Project. (**F**) Heatmap of linkage disequilibrium (LD) values (D' and r²) across the 14 SNPs in the ATP2B4 gene. The color gradients indicate the degree of association (D') and correlation (r²) between each pair of SNPs.

Several SNPs of ATP2B4 gene have been studied for their association with malaria protection. This study focusses on SNPs selected on the basis of either strong GWAS signal or experimental research (Table S1). These SNPs are clustered in three regions of the gene: Enhancer (1st intron), 5′UTR, and second intron (Fig. 1A). According to the previous studies, alleles of these SNPs have been categorized as SMPA (severe malaria protective allele), SMSA (severe malaria susceptible allele), MMPA (mild malaria protective allele), and MMSA (mild malaria susceptible allele) (Table S1). This categorization is based on their association with either severe/mild malaria protection or susceptibility.

Using the UCSC genome browser, regulatory mechanisms like histone modification, DNase hypersensitive region, and remap density plot were explored for these regions. The first intronic region acts as *cis*-regulatory elements (cCREs), likely regulating PMCA expression. The 5′UTR shows promoter like activity and is predicted to serve as a secondary promoter specific to erythrocytes. However, the second intronic region, despite being associated with malaria protection in GWAS, did not exhibit any clear involvement in regulatory mechanism (Fig. S1).

Variants of ATP2B4 gene were explored in the Indi Genomes (Indian genome database) (52), which shows the frequency of altered alleles for all SNPs ranges from 0.8 to 0.9 in the Indian population (Table S1). Genotyping in 117 random malaria negative apparently healthy individuals identified 14 SNPs in the same regulatory regions (Table S1; Fig. 1A, indicated by star shape). According to the Hardy-Weinberg test result all the analyzed SNPs are in Hardy-Weinberg equilibrium (Table S2). Genotyping of these variants shows allelic frequency data corroborate with Indian database comprising the genotype data of 1,000 healthy Indian individuals (Fig. 1B and C; Table S3). Further investigation was extended to various malaria outcomes in other studies to check the frequency of these variations in *P. falciparum* positive and severe malaria cases.

Further, genotyping of 14 SNPs of ATP2B4 gene was performed in severe malaria and mild malaria cases. Six SNPs were observed in enhancer region which yield three genotypes: TACTTC/TACTTC, CTTCCT/CTTCCT, and heterozygous for all. In the 5′UTR, five SNPs were observed which yield three different genotypes: CCGAG/CCGAG, TTCGA/TTCGA, and heterozygous for all. Similarly, in the 2nd intron, 3 SNPs were observed and yield three genotypes: TAC/TAC, GGT/GGT, and heterozygous. All the observed genotypes of studied regulatory regions showed equivalent prevalence between healthy group, *P. falciparum*-negative, *P. falciparum*-positive, and severe malaria samples (Fig. 1B; Table S3), indicating that genetic variations in regulatory regions of ATP2B4 are not associated with different malaria outcomes.

### The Indian population exhibit distinct pattern of linkage disequilibrium compared with global population

All the studied 14 SNPs are in 3 kb DNA fragment spanning the chromosome region chr1:203681464-203684970 (Fig. 1A). Haplotype analysis of all 14 SNPs identified four haplotypes in LD-link (a web-based application that analyzes the linkage pattern in populations of 1000 Genomes Project) using all population groups from the world. In total, six haplotypes were observed in the studied Indian population with various frequencies. Every haplotype observed in the Indian population displayed a mosaic pattern of alleles that were previously reported as protective or susceptible against severe malaria. Likewise in major haplotype, previously reported SMPA/MMPA (indicated by green color) were observed for enhancer region while previously reported SMSA/MMSA (indicated by red color) were observed for 5′UTR and 2nd intron (Fig. 1D). This similar pattern was also observed in haplotypes created by the 1000 Genomes Project database (Fig. 1E).

LD plot of all the SNPs shows two blocks of variants present in complete linkage in Indian population, one having only promoter region and other one with 5′UTR and 2nd intron (Fig. 1F). However, similarly, in the LD plot from the 1000 Genomes Project the same set of variants displayed complete linkage among all the SNPs, suggesting a different LD pattern across the Indian population (Fig. S2).

### Effect of ATP2B4 polymorphisms on RBC indices, PMCA4b expression, basal calcium levels in RBCs, and *in vitro* parasite growth

**Fig 2:**
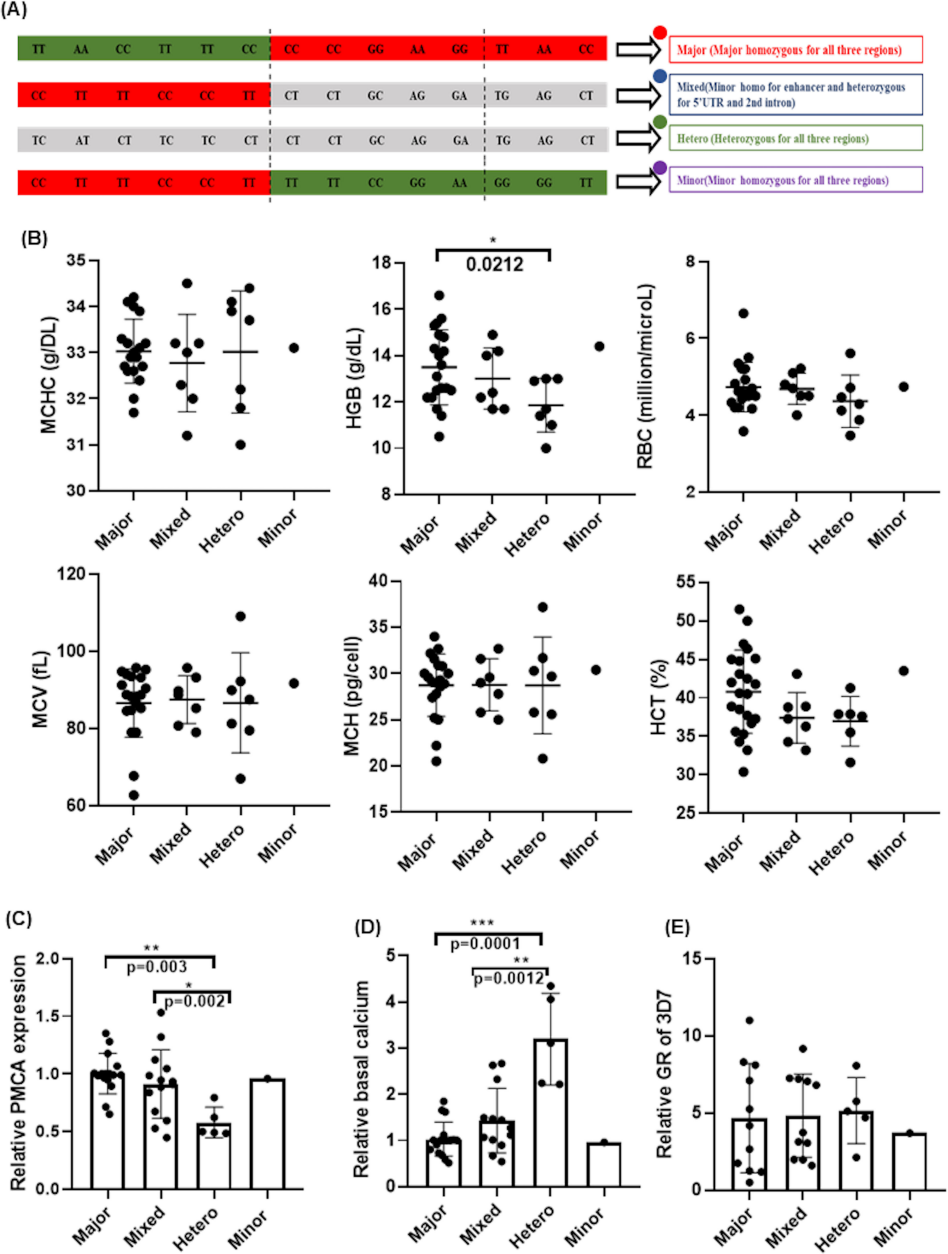
Effect of ATP2B4 gene variations on RBC indices, PMCA4b expression, basal calcium levels, and 3D7 growth rate in the studied Indian population. (**A**) Representation of the four ATP2B4 genotypes observed in the Indian population, selected for further analysis. (**B**) RBC indices representing MCHC (mean corpuscular hemoglobin concentration), HB (hemoglobin), RBC (red blood cell count), MCV (mean corpuscular volume), MCH (mean corpuscular hemoglobin), and HCT (hematocrit) in 4 ATP2B4 genotypes (*N* = 32, healthy donors). (**C**) PMCA4b expression levels (*N* = 34, healthy donors) and (**D**) basal calcium levels (*N* = 34, healthy donors) across the four ATP2B4 genotypes. Both measurements are plotted with mean fluorescence intensity (MFI) on the y-axis and genotypes on the x-axis. *P*-values are indicated in the figures. (**E**) 3D7 growth rate across different genotypes (*N* = 28, healthy donors). Asterisks denote statistical significance (*P < 0.05, **P < 0.01, ***P < 0.001).

A total of four genotypes were observed in the studied population, representing a combination of alleles for all the 14 SNPs (Fig. 2A): major, mixed, heterozygous, and minor. The effects of these genotypes on RBC indices, PMCA4b expression, and basal calcium level were investigated. The bar graphs show the effect of ATP2B4 genotype on hemoglobin (Hb), mean corpuscular volume (MCV), mean corpuscular hemoglobin concentration (MCHC), mean corpuscular hemoglobin (MCH), hematocrit (HCT), and red blood cell count (Fig. 2B). The result suggests no significant association of any genotype with RBC indices other than Hb level (*P* = 0.0212). The heterozygous genotype shows a significantly low level of Hb compared with the major genotype.

PMCA4b expression was analyzed by flow cytometry; however, to check the specificity of anti-PMCA4b antibody, western blotting was performed. A distinct ~130 kDa band corresponding to PMCA4b was detected in RBC lysates but absent in parasite lysates, confirming the antibody’s specificity for host-derived PMCA4b (Fig. S3). Furthermore, analysis of PMCA4b expression in erythrocytes of different genotypes was performed using flow cytometry. The graph (Fig. 2C) shows relative PMCA4b expression levels across four genotypes. These data indicate significant differences in PMCA expression among these genotypes by using Kruskal-Wallis tests (*P* = 0.0051). Furthermore, Dunn’s multiple comparisons test shows that heterozygous genotypes have significantly lower PMCA4b expression than major and mixed genotypes. Basal calcium levels were also investigated among these four genotypes (Fig. 1G). Kruskal-Wallis test indicates significant difference in calcium level among these genotypes (*P* = 0.0034). Further, Dunn’s multiple comparisons test shows that the heterozygous genotype has significantly higher basal calcium level than major and mixed genotypes (Fig. 2D). The minor genotype displays a higher PMCA4b expression level and lower calcium level, although no statistical comparison could be conducted because we had only a single sample. *In-vitro* parasite growth assays were done to assess the malaria susceptibility of different genotypes. However, no significant changes in the growth of parasite in different ATP2B4 genotypes were observed (Fig. 2E). The phenotypic difference in the heterozygous individuals suggests that heterozygosity in the enhancer region plays a significant role in PMCA4b expression and resulting basal calcium levels. This is supported by the fact that individuals with a mixed genotype (homozygous minor for the enhancer and heterozygous for the 5’UTR and second intron) differ only in the enhancer region compared with heterozygous individuals and do not show variations in other phenotypes.

### Inverse association of PMCA4b expression with intraerythrocytic calcium levels

**Fig 3:**
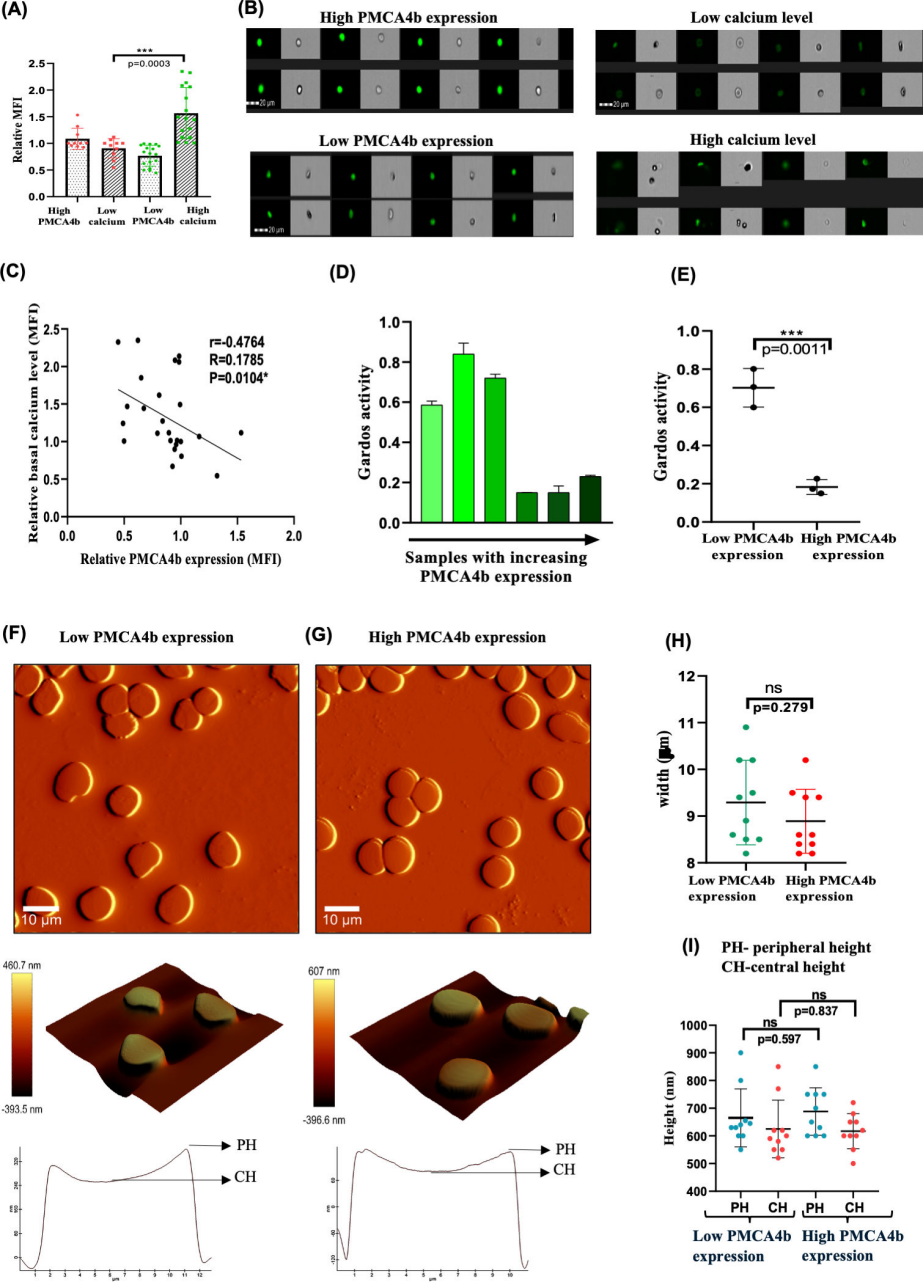
Impact of PMCA4b expression on red blood cell morphology and structural measurements. (**A**) Stacked column graph depicting the relationship between high and low PMCA4b expression and basal calcium levels. (**B**) Representative images from imaging flow cytometry illustrating high and low PMCA4b expression and corresponding low and high calcium levels. (**C**) Scatter plot demonstrating the inverse correlation between PMCA4b expression and calcium concentration. Spearman correlation coefficient and *P*-values are indicated in the figure (*N* = 28, healthy donors). (**D**) Measurement of Gardos channel activity in RBCs with different PMCA4b expression (expression in increasing order on x-axis) assessed using the FluxOR Potassium Ion Channel Assay (*N* = 6, healthy donors). Fluorescence intensity was measured before stimulus, which is considered basal level, and after stimulus. Stimulation with thallium (TI) is plotted on the y-axis, indicating potassium flux through the Gardos channel. (**E**) Combined activity of the Gardos channel in RBCs with high and low PMCA4b expression levels. *P*-value indicating significance is indicated. (**F**) Atomic force microscopy (AFM) images of RBC with low PMCA4b expression. Scale = 10 µm. (**G**) AFM image of RBC with high PMCA4b expression (scale = 10 µm), illustrating surface characteristics and overall morphology reflective of the physiological state associated with PMCA4b levels. Zoomed AFM image (scale = 6 µm), showcasing detailed structural features including roughness and height profiles that highlight the microtopography of RBCs with PMCA4b levels. (**H**) Width and (**I**) height measurements of red blood cells (RBCs) with respect to PMCA4b levels are plotted. Asterisks denote statistical significance (*P < 0.05, **P < 0.01, ***P < 0.001).

To investigate the relationship between PMCA4b expression and basal calcium level, samples were divided into high and low PMCA4b expression groups using the median relative MFI value across all donors as the cutoff. Samples with expression values greater than or equal to the median were classified as “high,” whereas those below the median were classified as “low.” The average basal calcium levels in each group, measured using Fluo-4 AM, were compared, which revealed significant a difference between the two categories (*P* = 0.0003) (Fig. 3A). Representative imaging flow cytometry images also illustrate high calcium staining in low PMCA expressing cells and vice versa, indicating the reverse correlation (Fig. 3B).

To nullify the efflux of Fluo-4AM by erythrocyte transporters, control assays were performed where Fluo-4 AM was loaded in erythrocytes with or without probenecid (Fig. S5) at two time points (3 h and 6 h). The results confirmed that data acquisition up to 3 h after Fluo-4AM loading does not demonstrate any significant difference between probenecid treated and untreated samples. However, at 6 h time point, there is fluorescence difference between probenecid treated and untreated samples, indicating the loss of dye during prolonged measurements (Fig. S5).

Following this observation, we analyzed the linear correlation of PMCA4b expression with intraerythrocytic calcium levels, and a linear regression test was performed among the samples irrespective of their genotypes. The regression analysis reveals an inverse correlation between PMCA expression and intracellular basal calcium level in RBCs (negative slope of −0.8664, 95% CI). Additionally, Spearman’s correlation coefficient supports this correlation (*P* = 0.0104) (Fig. 3C). This observation suggests that PMCA4b expression inversely correlates with basal calcium levels in RBCs.

### Low PMCA4b expression cause the activation of Gardos channel but does not affect the RBC hydration status or morphology

Elevation of intraerythrocytic calcium levels activates Gardos channels to efflux potassium ions outside RBCs. Gardos channel activation was investigated by FluxOR potassium ion channel assay. This assay measures fluorescence intensity, indicating potassium flux through the Gardos channel. Gardos channel activation was assessed at basal levels (without stimulus) and after stimulation with thallium (Tl). The fluorescence intensity of each sample was normalized with an average baseline fluorescence intensity measured before adding stimulus (TI). The result shows that RBCs with high PMCA expression (lower basal calcium level) exhibit less activity of the Gardos channel than RBCs with low PMCA expression (Fig. 3D). The activity of the Gardos channel is directly influenced by basal calcium levels, with fluorescence intensity decreasing as basal calcium levels decrease (Fig. 3E). The difference was statistically significant (*P* = 0.0011), indicating the role of PMCA4b expression in regulation of Gardos activity.

Activation of Gardos channels facilitates echinocytosis of RBCs. To observe echinocytic RBCs under variable intraerythrocytic calcium levels, side scatter (SSC) analysis was performed using flow cytometry. SSC values correspond to cell granularity and hydration status, with increasing SSC of erythrocytes suggesting cell dehydration. Pearson’s correlation test indicates a positive trend between basal calcium level and SSC values; however, this correlation was not statistically significant (*P* = 0.0695) (Fig. S4), suggesting no association of PMCA4b derived calcium with RBC dehydration.

We further performed atomic force microscopy (AFM) to examine in detail the morphological and structural changes in RBC with high and low PMCA level. In total, 2D and 3D AFM images of RBCs with low (Fig. 3F) and high (Fig. 3G) PMCA expression showed no significant changes in surface features. In addition to lack of visual morphological differences, quantitative measurement of RBC width and height was also analyzed to determine if PMCA level influence the RBC size. Width (Fig. 3H) and height (Fig. 3I) results showed no statistically significant difference (*P* = 0.597 for peripheral height, *P* = 0.837 for central height). Similarly, there was no statistically significant difference in the width of RBCs with high and low PMCA level (*P* = 0.279).

### Expression of PMCA4b on erythrocytes positively associates with *P. falciparum* growth and invasion

**Fig 4:**
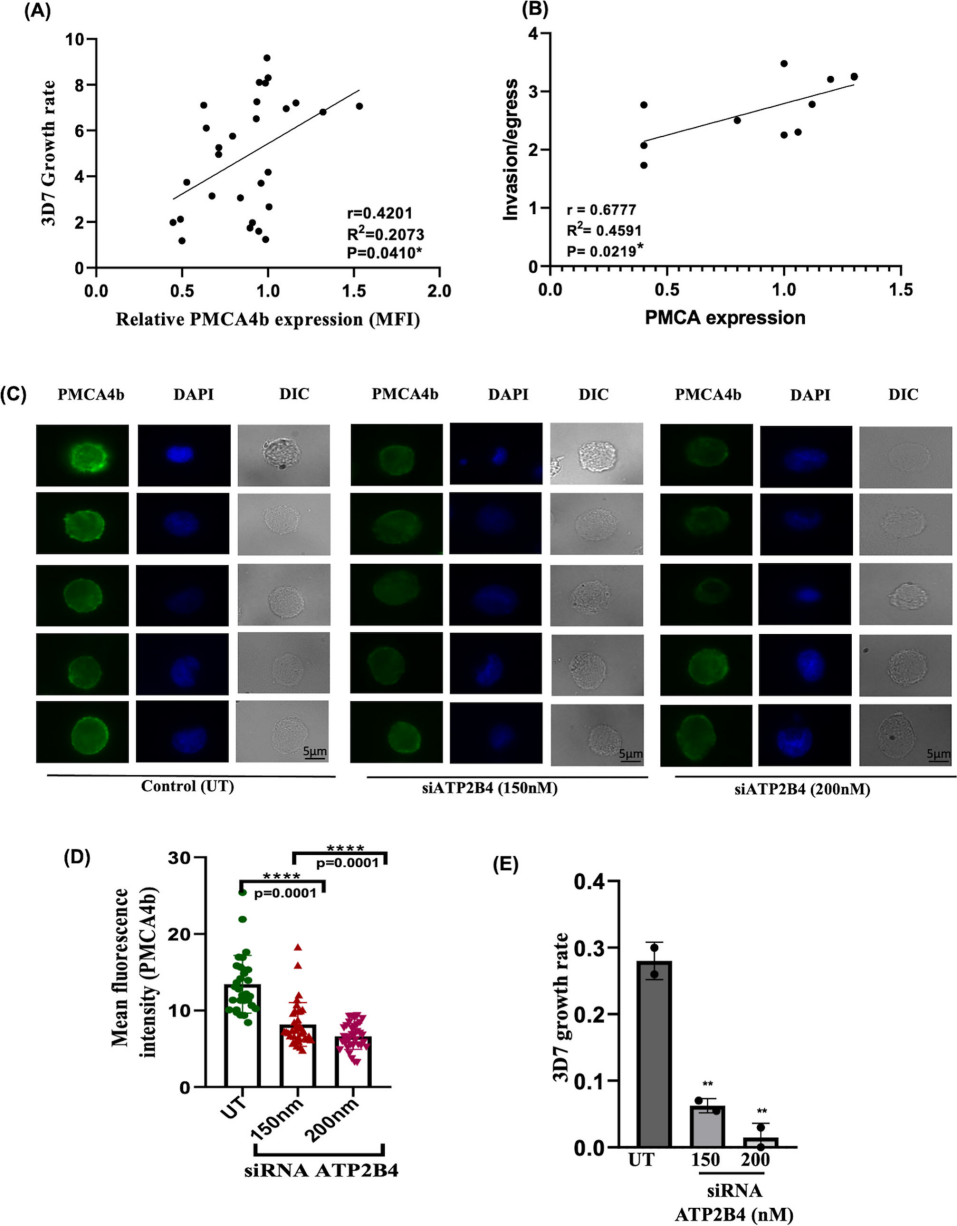
Effect of PMCA4b expression on 3D7 growth in donor RBCs and siRNA-mediated PMCA inhibition. Effects on calcium levels, ROS production, and *P. falciparum* growth in BEL-A cells. (**A**) Scatter plot showing the direct relationship between PMCA4b expression levels and the growth rate of the *P. falciparum* 3D7 strain. Lower PMCA4b levels are associated with decreased parasite growth. Spearman correlation coefficient and *P*-values are indicated in the figure (*N* = 28, healthy donors). (**B**) Effect of PMCA4b expression in invasion per egress of 3D7 (*N* = 10, healthy donors). (**C**) siRNA mediated silencing of ATP2B4 gene in BEL-A cells (Bristol Erythroid Line Adult cells). Representative fluorescence microscopy images show PMCA4b expression analyzed by fluorescence microscopy. (**D**) Mean fluorescence intensity (MFI) analysis of PMCA expression in untreated (UT), 150 nM siRNA-treated, and 200 nM siRNA-treated cells, indicating effective reduction of PMCA expression with increasing siRNA concentration. (**E**) *P. falciparum* growth rate in UT and siRNA-treated cells (150 nM and 200 nM), showing a potential impact of PMCA downregulation on parasite growth. Asterisks denote statistical significance (*P < 0.05, **P < 0.01, ***P < 0.001).

Previous studies correlated ATP2B4 SNPs with malaria protection, but this study found no significant difference in the parasite growth in RBCs from different ATP2B4 genotype except for the heterozygous individuals who are consistent with low PMCA4b expression. Irrespective of the genotype, PMCA4b expression has been seen to vary in the studied population. Hence, the effect of PMCA4b expression on intraerythrocytic growth of *P. falciparum* was monitored. Briefly, purified schizonts were incubated with RBCs having variable PMCA4b expression, and parasite growth was monitored after 48 h. The result shows a significant positive relationship between PMCA4b expression and growth rate of malaria parasite, indicating that low PMCA4b expression may confer protection from malaria (Fig. 4A). We further observed significant association of parasite invasion with varying levels of PMCA4b expression on RBCs (Fig. 4B).

### Downregulation of ATP2B4 expression by siRNA mimics the growth inhibition effect observed in individuals with naturally low PMCA4b levels

To confirm that reduced PMCA4b expression is an important factor in parasite growth, ATP2B4 mRNA was downregulated using siRNA in Bel-A cells (erythrocyte progenitor cells). A decrease in PMCA4b expression was observed following transfection of 150 nM and 200 nM of ATP2B4 targeting siRNA (Fig. 4C and D). Statistical analysis using one-way ANOVA with Tukey’s multiple comparisons test revealed a significant difference among the groups (*P* < 0.0001). Specifically, PMCA4b expression was significantly lower in cells treated with 150 nM siRNA (mean MFI = 8.18, *P* = 0.0001) and 200 nM siRNA (mean MFI = 6.64, *P* = 0.0001) than in the untreated control group (mean MFI = 13.44). However, the difference between the 150 nM and 200 nM siRNA groups was not statistically significant (*P* = 0.0632) at 95% CI. Additionally, inhibition of PMCA4b via siRNA at both 150 nM and 200 nM concentrations corresponded to a notable reduction in the growth rate of *P. falciparum* 3D7 (Fig. 4E), confirming the previous observation that lower PMCA4b levels negatively impact parasite growth within RBCs.

### Chemical inhibition of PMCA4b activity by resveratrol and ATA also reproduced the growth inhibition effect

**Fig 5:**
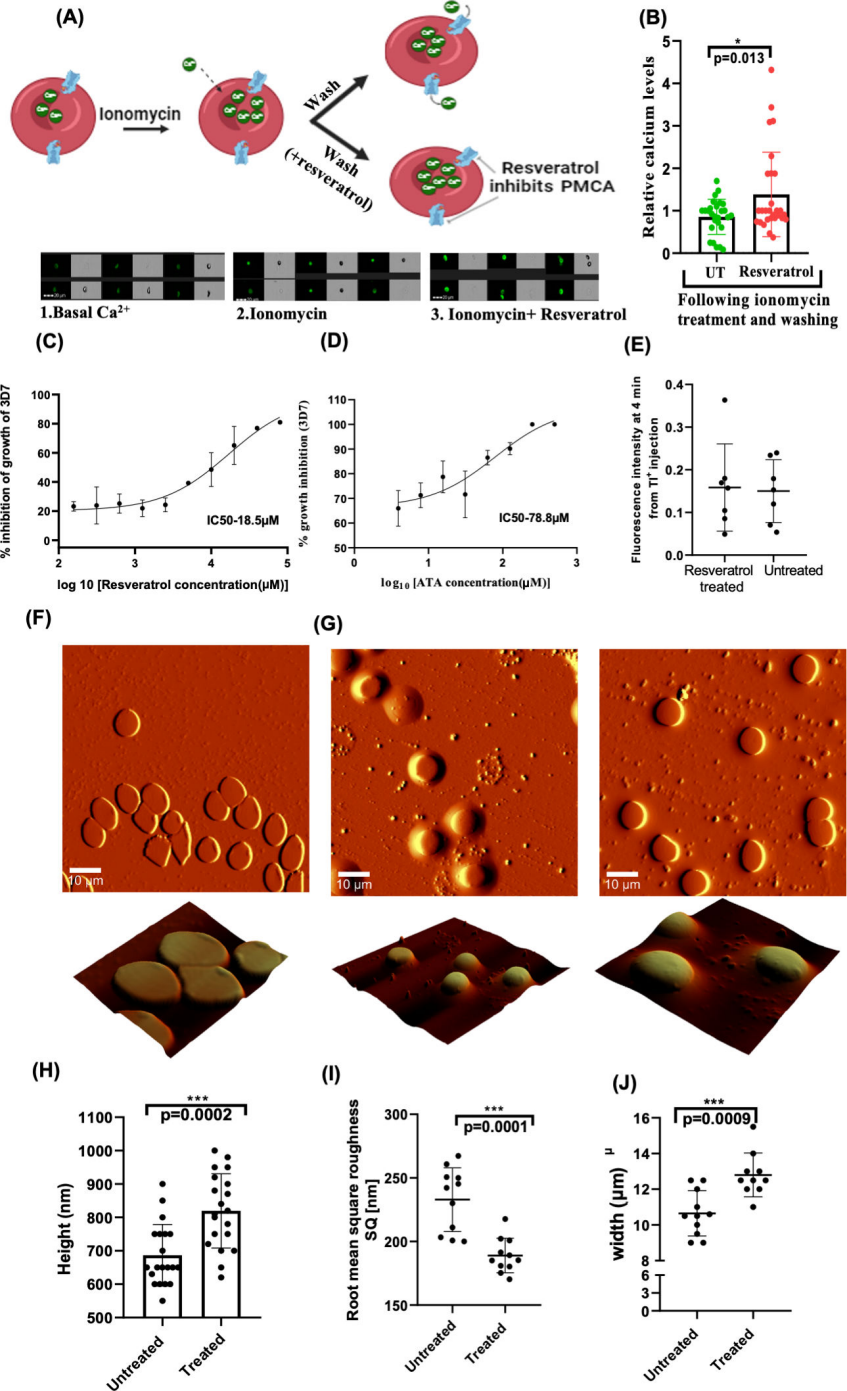
Resveratrol-mediated PMCA inhibition. Effects on calcium levels, ROS production, *P. falciparum* growth, Gardos channel activity, and morphology in donor RBCs. (**A**) Schematic diagram of the calcium analysis in RBCs, along with representative fluorescence images. RBCs were treated with ionomycin in calcium-containing medium to induce calcium influx, and basal fluorescence was measured. Following a 2 h treatment with resveratrol (50 µM), fluorescence was measured again to assess the effect of PMCA inhibition on calcium efflux. (**B**) Effect of resveratrol on intracellular calcium levels in RBCs. The observed fluorescence intensity in ionomycin serves as basal calcium level, and fluorescence in RBC with ionomycin + resveratrol reflects changes in calcium levels after PMCA inhibition. *P*-values are indicated in the figure (*N* = 28). (**C**) Effect of PMCA4b inhibition by resveratrol and (**D**) ATA on parasite growth. Growth inhibition assay (GIA) curves show the antimalarial effects of resveratrol and ATA on parasite growth after 48 hours of treatment. (**E**) Effect of PMCA4b inhibition by resveratrol on gardos channel activity was measured by FluxOR potassium ion assay. Potassium ion efflux was measured with and without treatment of RBCs with resveratrol, and fluorescence intensity was measured at 4 min after adding stimulus (*N* = 7). (**F**) Atomic force microscopy images of RBC before treatment and (**G**) after treatment with resveratrol (scale = 10 µm), illustrating surface characteristics and overall morphology reflective of the physiological state associated with PMCA4b inhibition by resveratrol treatment. Zoomed AFM image (scale = 6 µm), showcasing detailed structural features. Graph showing effect of PMCA4b inhibition on height (**H**), roughness (**I**), and width (**J**) profiles that highlight the microtopography of RBCs before and after treatment.Asterisks denote statistical significance (*P < 0.05, **P < 0.01, ***P < 0.001).

Previous results indicate that low PMCA4b expression correlates with increased calcium level and reduced *P. falciparum* growth. To further validate this observation, intraerythrocytic calcium levels and parasite growth were monitored following the inhibition of PMCA4b by resveratrol (Fig. 5A). Resveratrol is a naturally occurring polyphenol with pleiotropic biological effects, and it has been shown to inhibit PMCA activity in certain cell types (53). Similarly, aurintricarboxylic acid (ATA) is a broad-spectrum compound known to inhibit PMCA activity (54). These compounds as pharmacological inhibitors of PMCA4b have shown anti-malarial effects (55). Comparative analysis of calcium levels was performed in the presence and absence of resveratrol. Calcium levels were significantly high in resveratrol treated RBCs, indicating the inhibition of calcium efflux (mean difference: 0.5276 ± 0.2069; 95% CI: 0.1126 to 0.9425). An unpaired *t*-test confirmed this difference to be significant (*P* = 0.0137) (Fig. 5B). Representative flow cytometry images illustrate basal calcium levels, calcium after ionomycin treatment, and calcium levels after treatment with ionomycin and resveratrol. A control experiment was performed where PBS and PBS + BSA washing was performed for complete removal of ionomycin. No significant difference was observed with respect to the type of washing done (Fig. S6). Although our data suggest that PBS washes can effectively reduce residual ionomycin, previous studies have noted that complete removal may require lipids or detergents in the washing buffer (45).

Furthermore, resveratrol demonstrated dose-dependent growth inhibition of *P. falciparum* parasites with an IC50 of 18.35 µM (Fig. 5C). Another inhibitor of PMCA4b, aurintricarboxylic acid (ATA), was also tested for parasite growth inhibition and demonstrated an IC50 of 78.8 µM (Fig. 5D). These results suggest that the inhibition of PMCA4b restricts parasite growth, underscoring its potential as a druggable target. These finding are consistent with parasite growth pattern in field samples with variable PMCA4b expression.

### Effect of inhibition of PMCA4b on RBC dehydration or morphology

The effect of resveratrol treatment on Gardos channel activation was investigated, and it was found that Gardos activation is not influenced by PMCA4b inhibition (Fig. 5E). Atomic force microscopy (AFM) was used to analyze the morphological and structural changes in resveratrol-treated RBCs. Both 2D and 3D AFM images revealed significant alterations in RBC morphology after treatment (Fig. 5G) compared with untreated controls (Fig. 5F). Measurements of height, width, and surface roughness (SQ) were conducted and analyzed using *t*-test. Height (*P* = 0.0002) and width (*P* = 0.0009) increased significantly following treatment, indicating a marked swelling of RBCs. Additionally, surface roughness decreased significantly (*P* = 0.0001), further supporting the observation of overhydration in treated RBCs (Fig. 5H through J). Our data indicate that PMCA4b inhibition by resveratrol caused the RBCs to swell rather than dehydrate. This contradictory observation of RBC overhydration in the presence of elevated intracellular Ca²^+^ may reflect the emerging model of Gardos channel regulation proposed in a recent study (56), which showed that PMCA4b directly interacts with and regulates the Gardos channel (KCNN4) in RBCs via its C-terminal domain, independently of calcium extrusion activity, and that this inhibition is conformation-dependent, which can remain unaffected by pharmacological inhibitors of PMCA4b.

### Host PMCA4b-associated redox imbalance alters parasite growth and sensitivity toward artemisinin

**Fig 6:**
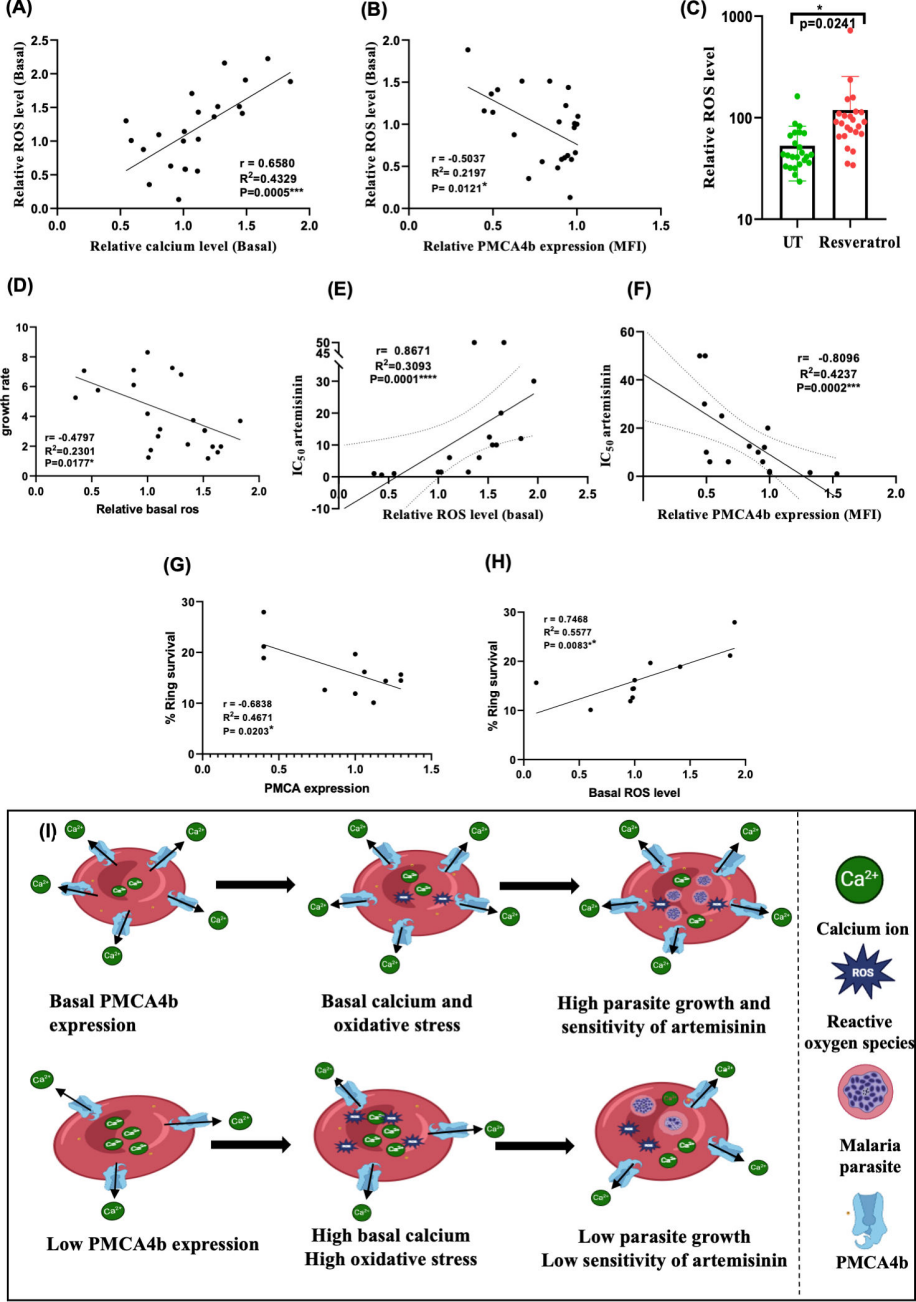
Role of oxidative stress of RBCs and PMCA4b expression in modulating artemisinin eEfficacy in *P. falciparum*. (**A**) Scatter plot illustrating a direct relationship between ROS and calcium levels in RBCs, suggesting that elevated calcium levels correlate with higher ROS production (*N* = 24, healthy donors). (**B**) Scatter plot showing the inverse relationship between PMCA4b expression and ROS levels in RBCs, measured using DCFDA dye. Lower PMCA4b levels correlate with higher ROS production (*N* = 24, healthy donors). (**C**) Effect of resveratrol on ROS levels in RBCs. Following a 2 h treatment with resveratrol (50 µM), cells were stained with DCFDA dye, and ROS levels were measured using flow cytometry (*N* = 24, healthy donors). (**D**) Effect of ROS level on growth rate of 3D7(*N* = 21, healthy donors). (**E**) Scatter plot illustrating the inverse relationship between ROS levels and artemisinin sensitivity, measured as IC₅₀ of DHA. (**F**) Scatter plot showing the direct relationship between PMCA4b expression levels and artemisinin sensitivity (IC₅₀). Spearman correlation coefficient and *p*-values are indicated in the figure. (**G**) Scatter plot showing inverse relationship of percentage ring survival of *P. falciparum* and PMCA4b expression (**H**) Scatter plot showing direct relationship of ROS level and percentage ring survival of *P. falciparum* infecting different PMCA4b expressing RBCs. (**I**) Final hypothesis model showing the relation between surface PMCA4b expression of RBCs, calcium levels, oxidative stress, *P. falciparum* growth, and artemisinin sensitivity. Asterisks denote statistical significance (*P < 0.05, **P < 0.01, ***P < 0.001).

High cellular calcium is often linked with high ROS levels. We further investigated the relationship between calcium and oxidative stress inside the RBCs using DCFDA dye as an indicator of ROS (reactive oxygen species). Control experiment using probenecid showed no significant difference in the fluorescence in the presence or absence of probenecid immediately after loading. However, fluorescence was slightly higher in the presence of probenecid, but relative differences across studied samples remained consistent (Fig. S5B). Linear regression analysis showed a significant positive correlation between calcium and ROS level, indicating that increased calcium level correlates with higher ROS level. Further, Pearson’s correlation coefficient confirmed this correlation to be significant (*P* = 0.0005) (Fig. 6A). Similarly, the inverse relationship between PMCA4b level and ROS level was analyzed. Pearson correlation coefficient confirmed this negative correlation (*P* = 0.0121) (Fig. 6B), suggesting that reduced PMCA4b expression level leads to increasing calcium concentration, which contributes to increased oxidative stress in RBCs. To further confirm that the change in ROS levels was due to PMCA4b polymorphism but not any other variation, the impact of resveratrol treatment on ROS production in RBCs was investigated. A paired *t*-test comparing basal ROS levels and ROS levels following resveratrol treatment indicates a statistically significant increase in ROS generation after resveratrol exposure (*P* = 0.0355). The mean difference in ROS levels between the two conditions was 77.53, with a 95% CI of 5.861–149.2 (Fig. 6C), indicating the specific role of PMCA4b in redox imbalance. To eliminate the potential confounding effects on RBC redox biology, donors used in oxidative stress assays were screened for G6PD deficiency and hemoglobinopathies (Table S5), and blood samples from those having normal G6PD activity and HbAA hemoglobin were used in the assay.

Oxidative stress in RBCs has been implicated in inhibiting malaria parasite growth. To explore this relationship, the intraerythrocytic growth rate of the parasite was correlated with RBC oxidative stress levels using Pearson correlation analysis. The analysis revealed a significant negative correlation (*P* = 0.017), indicating that higher oxidative stress in RBCs is associated with reduced parasite growth. (Fig. 6D). Contrary to previous studies suggesting dehydration as a protective mechanism against malaria, we could not find any association of dehydration with parasite growth. Rather, our data indicate the involvement of oxidative stress as the protective mechanism.

Oxidative stress of host erythrocytes predisposes the malaria parasites to develop tolerance against oxidative damage-causing drugs such as artemisinin. The correlation analysis between the ROS levels of RBCs and antimalarial IC_50_ of artemisinin shows a positive slope, indicating the loss of artemisinin sensitivity in the presence of redox imbalance. The regression analysis was statistically significant with a *p*-value of 0.0001 (F = 6.717), suggesting that the slope is significantly different from zero. The correlation coefficient (r) of 0.8671 (95% CI: 0.1030–0.8181) supports a positive association, with an R-squared value of 0.3093. These results suggest that increases in the ROS level of RBCs are associated with higher artemisinin IC_50_ values (Fig. 6E).

The corresponding relationship between PMCA4b expression levels of RBCs and *P. falciparum* sensitivity toward artemisinin was also investigated. A significant inverse correlation was observed between PMCA4b level and IC_50_ of artemisinin, as represented by the linear regression analysis, indicated that lower PMCA4b levels in RBCs were associated with increased IC_50_ of artemisinin in the parasite. The slope of the regression line was −33.35 (95% CI: −54.75 to −11.94), significantly different from zero (F = 11.03, *P* = 0.0002), suggesting that variations in PMCA4b level have a measurable impact on artemisinin efficacy. Correlation coefficient (r = −0.8096) further confirmed a significant negative correlation, with a 95% CI of −0.8619 to −0.2478 (Fig. 6F).

The decreased sensitivity of artemisinin in parasites growing within RBCs under high oxidative stress was further validated using the ring survival assay. The results revealed a high percentage of ring survival in RBCs with low PMCA4b expression (Fig. 6G) and elevated oxidative stress levels (Fig. 6H), substantiating a strong link between oxidative stress and reduced artemisinin efficacy. Furthermore, heterozygous ATP2B4 genotype demonstrated higher IC50 of artemisinin than other genotypes, again implying that low PMCA4b expression leads to reduced artemisinin efficacy (Fig. S7). These data suggest a new concept of host-mediated evolution of drug resistance, particularly in the areas where several forms of ROS-inducing hemoglobinopathies are prevalent.

## DISCUSSION

Artemisinin-based combination therapies are the frontrunners in malaria treatment and have played a major role in the malaria elimination program. Emergence of resistance in parasite against artemisinin or other partner drugs has caused a serious threat in the global malaria elimination efforts. The reduced sensitivity of *P. falciparum* to artemisinin is caused by the mutations in the propeller domain of the kelch13 gene (PfK13) inducing dormancy and reduced metabolic activity in the parasites until the serum drug concentration is reduced. Some studies also suggest role of stress response pathways and modulation in endocytosis of parasite. The active surveillance of mutations in K13 locus is being done to track artemisinin resistance. However, delayed parasite clearance has also been reported without PfK13 mutations, indicating the role of other factors in development of artemisinin resistance (9, 57). Recent studies also indicated a role of WD40 and RAD5 in addition to K13 locus in delayed parasite clearance (58, 59).

The parasite demonstrates a close relationship with its human host; hence, parasite biology cannot be studied independent of the host physiology. A number of studies have indicated the role of host proteins such as Band 3, Glycophorins, and Basigin as a crucial factor for parasite infection (60, 61). RBC calcium channels also play an important role in this host-parasite interaction. Host Gardos channel (calcium activated potassium channel) inhibition has potent antimalarial activity, indicating its role in disease pathogenesis (62); PIEZO, a mechanosensitive calcium channel, has been also studied for its role in malaria protection (63). Additionally, polymorphisms in the human ATP2B4 gene, encoding a calcium channel, are associated with malaria protection (19). At the genetic level, multiple single-nucleotide polymorphisms (SNPs) in the regulatory regions of ATP2B4 confer protection against severe and mild malaria (19, 20, 22, 23, 27, 64, 65). Mostly, the studies were focused on African populations, often overlooking the southeast Asian populations where both *P. falciparum* and *P. vivax* malaria are endemic.

This study was rationally designed to correlate both field and experimental data. It identified that the previously reported SNPs in ATP2B4 regulatory region are common in studied Indian population, as well in Indigenome database, but the protection against severe/mild malaria cannot be seen in studied samples. This could be attributed to different genetic makeup of Indian population and African population, where most of the ATP2B4 studies were conducted, suggesting that population-based host genetic differences have a huge influence on malaria protection. Additionally, our study does not strongly state that there is no association; it points out the possibility that a true effect could exist but was not detected due to the limited samples. Haplotype analysis suggests that major haplotypes present in Indian population (also in 1000 Genomes Project populations) have both malaria protective and malaria susceptible alleles; this complexity of genetic makeup makes it important to dissect how individual alleles influence malaria susceptibility. Additionally, ATP2B4 genotypes do not associate strongly with the PMCA4b expression and basal calcium levels across the studied population. However, individuals heterozygous for all SNPs exhibit significantly low PMCA4b expression, high basal calcium, and decreased hemoglobin (Hb). These findings highlight the critical role of enhancer region in regulating PMCA4b expression and other RBC physiology, as individuals with mixed genotype, differing only in enhancer region, do not exhibit these physiological alterations. Further variations present in enhancer region only show their effect in heterozygous state, suggesting a dosage-dependent regulatory mechanism for GATA-1 binding (66). Also, PMCA4b expression was found to vary among individuals independent of the ATP2B4 genotypes, indicating the involvement of additional regulatory factors beyond genetic variation at this locus; whereas ATP2B4 variations have previously been hypothesized to confer malaria protection by reducing PMCA4b expression, this effect could not be confirmed experimentally in the studied population.

In line with earlier reports showing no association between ATP2B4 genotype and merozoite invasion (26), our study demonstrates that it is the expression level of PMCA4b, rather than the genotype itself, that influences parasite invasion and growth. Our finding that RBCs with low PMCA4b expression exhibit reduced invasion is consistent with the recent report by Jamwal et al. (67), which demonstrated that basigin, the essential receptor for *P. falciparum* invasion, exists in macromolecular complexes with PMCA/4 or MCT1. Importantly, PfRH5 interacts with these basigin/PMCA complexes with higher affinity than with basigin alone, highlighting the physiological relevance of PMCA in invasion. Thus, reduced PMCA4b expression may alter the abundance or stability of basigin/PMCA complexes, thereby contributing to the impaired invasion phenotype we observed. Although the invasion assay was performed with a relatively small number of donor samples, the consistent trend and statistical power observed between PMCA4b expression and reduced parasite invasion support the robustness of our findings.

Furthermore, we observed that PMCA4b expression strongly correlated with basal intraerythrocytic calcium levels. Individuals with lower PMCA4b expression exhibit higher basal calcium and reduced parasite growth compared with those with higher PMCA4b expression. As hypothesized previously (26, 29, 64, 68), the low growth of *P. falciparum* may be attributed to calcium-mediated activation of Gardos channel, which induces Gardos effect, leading to efflux of potassium ions in RBCs with low PMCA4b. However, in this study detailed analyses by AFM did not reveal significant structural or morphological changes in RBCs with low PMCA4b expression. Additionally, SSC analysis also rules out the dehydration in these RBCs. This suggests that naturally occurring variations in PMCA4b expression might affect RBC physiology but their impact may not result in detectable alterations in RBC morphology or hydration status under normal conditions. To further investigate the effect of PMCA4b on RBC morphology, cells were treated with resveratrol. Interestingly, the inhibition of PMCA4b did not lead to dehydration of RBCs. This observation supports the recently hypothesized regulatory mechanism of gardos channel (56), where the role of PMCA4b C-terminus is indicated in the regulation of Gardos channel activity instead of the previously hypothesized intraerythrocytic calcium.

Additionally, we observed that high calcium levels were positively correlated with erythrocytic ROS levels as reported in previous studies for nucleated cells (16). Thus, the malaria-protective mechanism of RBCs with lower PMCA4b expression could not be attributed to changes in RBC hydration state; rather, changes in intraerythrocytic calcium and ROS levels could be a determining factor in the malaria protective mechanism of RBCs with variable PMCA4b expression.

The role of PMCA4b expression in *P. falciparum* growth was further supported by resveratrol-mediated inhibition of PMCA4b in RBCs and siRNA-mediated knockdown in BEL-A cells. Resveratrol increased intracellular calcium and demonstrated potent antimalarial activity (55). Similarly, in BEL-A cells, silencing of PMCA4b significantly suppressed parasite growth, highlighting its critical role in facilitating *P. falciparum* survival. These findings underscore the therapeutic potential of targeting PMCA4b as a strategy to combat malaria.

The study by Pires et al. identified that parasite survival mechanism to oxidative stress is similar to the artemisinin stress; thus, *P. falciparum* parasites growing under oxidative stress conditions can adapt and become less sensitive to artemisinin (12). In this study, intraerythrocytic ROS levels exhibit a strong positive correlation with calcium levels and negative correlation with PMCA4b expression, suggesting that RBCs with low PMCA4b expression and high calcium concentrations exhibit elevated ROS levels, creating a cellular environment that could influence parasite response towards artemisinin. This hypothesis is further supported by growth inhibition assays, which demonstrate that parasites growing in RBCs with lower PMCA4b level and higher calcium level become less sensitive to artemisinin, as indicated by an increase in IC50 values with increasing ROS levels and decreasing PMCA4b expression. Further ring survival assay in RBCs with different PMCA4b expression level suggested an increase in the percentage of ring survival in RBCs with lower PMCA4b and higher oxidative stress. These findings highlight the intricate interplay between RBC calcium homeostasis and oxidative stress in determining drug sensitivity of parasite surviving in such conditions, hence emphasizing the potential need to account for RBC physiological states when optimizing antimalarial treatments. These observations further call for an important future investigation to study the therapeutic efficacy of artemisinin in kelch-13 mutant parasites infecting low PMCA4b expressing RBCs.

Malaria endemic areas often see that selection of host genotypes such as hemoglobinopathies and G6PD deficiency, which naturally have high ROS levels, demonstrates some level of protection from malaria due to reduced parasite infection (69). However, once infected, parasites can be tolerant to ROS and hence to ROS-inducing drugs such as artemisinin as well. Thus, although evolutionary adaptations of the host may confer some amount of malaria protection, they may simultaneously make the host more vulnerable to drug resistance. Therefore, we propose that screening of host redox levels is crucial for planning containment and elimination strategies of drug-resistant malaria. Furthermore, the parasites demonstrated partial host-mediated drug resistance in our study, suggesting that doses of artemisinin administered can be modulated in individuals susceptible to drug-resistant malaria.

To conclude, this study highlights the critical role of host polymorphisms in modulating *P. falciparum* growth and sensitivity toward artemisinin. Lower PMCA4b expression is associated with reduced parasite growth; however, once the parasite survives under conditions of elevated ROS, it becomes more adaptive and exhibit decreased sensitivity to artemisinin. These findings suggest that while evaluating artemisinin resistance, we should not only focus on parasite variability but also consider the broader spectrum of interactions with human host factors, such as variations in erythrocyte environments. This comprehensive approach could provide deeper insights into host evolution against malaria infection as well as artemisinin resistance mechanisms and inform more effective intervention strategies.

## Data Availability

The raw data supporting the conclusions of this article will be made available by the authors, on request, to any qualified researcher.

## SUPPLEMENTAL MATERIAL

Supplemental material mbio.01738-25-s0001.pdf Supplemental figures and tables.

ASM does not own the copyrights to Supplemental Material that may be linked to, or accessed through, an article. The authors have granted ASM a non-exclusive, world-wide license to publish the Supplemental Material files. Please contact the corresponding author directly for reuse.
