# Supplementary material for: Decoding the multifaceted role of erythrocyte PMCA4b in oxidative stress-mediated malaria protection and artemisinin resistance
Source: mBio. 2025 Dec 16;17(1):e01738-25. doi: 10.1128/mbio.01738-25 (PMC12802218; doi:10.1128/mbio.01738-25)
Supplement: Supplemental material — Supplemental figures and tables. [file mbio.01738-25-s0001.pdf]

Supplementary material

Decoding the Multifaceted Role of erythrocyte PMCA4b in Oxidative stress mediated Malaria Protection and Artemisinin Resistance

Priya Agrohi[1,2, #], Swati Garg[3,4 #], Shreeja Biswas[3], Preeti Maurya[3], Vijay Kumar[1,2], Jyoti Sharma[3], Bidhan Goswami[5,6], Samir Kumar Sil[6], Mrigendra Pal Singh[7], Sunil Kumar Chand[7], Sneh Shalini[8], Om P Singh[1], Gunanidhi Dhangadamajhi[9], Neelima Mishra[1,2], Sivaprakash Ramalingam[10], Prashant Kumar Mallick* [1,2], Shailja Singh*[3]

[1] ICMR-National Institute of Malaria Research, New Delhi, India – 110077
[2] Academy of Scientific and Innovative Research (AcSIR) Ghaziabad, Uttar Pradesh, India- 201002
[3] Special Centre for Molecular Medicine, Jawaharlal Nehru University, New Delhi, India-110067
[4]Department of Life Sciences, Sharda University, Greater Noida, Uttar Pradesh, India- 201310
[5]Molecular Genetics and Cancer Biology Laboratory, Department of Human Physiology, Tripura University, Suryamaninagar, Tripura, India- 799130
[6]Department of Human Physiology, Tripura University, Suryamaninagar, Tripura, India- 799130

[7]ICMR-National Institute of Malaria Research Field Unit Jabalpur, Madhya Pradesh, India-482003
[8]Indian Council of Medical Research, New Delhi-India-110029
[9]Department of Biotechnology, North Orissa University, Baripada, Odisha, India- 757003
[10]CSIR-Institute of Genomics and Integrative Biology (CSIR-IGIB), New Delhi-110025
[#]Equal Contribution

*Corresponding authors

Correspondence: shailja.jnu@gmail.com, pkmmrc01@gmail.com

Table 1: Summary of severe/mild malaria protective/susceptible [SMPA/MMSA, MMPA/MMSA] alleles of ATP2B4 gene.

Table 2: Hardy Weinberg equilibrium analysis of 14 ATP2B4 SNPs.

Table 3: Prevalence of genotypes in Different Sample Groups.

Table 4: PCR condition and primers used for the amplification of regulatory DNA region of ATP2B4.

Table 5: Information of samples used in the study.

Figure 1: ATP2B4 regulatory regions identified by UCSC genome browser.

Figure 2: Linkage Disequilibrium plot of 14 SNPs of ATP2B4 across populations of 1000 genome project.

Figure 3: Validation of PMCA4b antibody specificity in RBCs.

Figure 4: Relationship between calcium level and SSC.

Figure 5: Effect of probenecid on dye retention in RBCs after dye loading

Figure 6: Effect of washing conditions (PBS vs. BSA) on Fluo 4AM loaded RBCs under different treatments.

Figure 7: Relationship between ATP2B4 genotypes and artemisinin sensitivity.

Table 1: Summary of the previously reported severe/mild malaria protective/susceptible [SMPA/MMSA, MMPA/MMSA] alleles of ATP2B4 gene.

This table provides a summary of malaria protective and susceptible allele based on previous research. The reference listed support the reported association. SMPA-severe malaria protective allele, SMSA-severe malaria susceptible allele, MMPA-mild malaria protective allele, MMSA- mild malaria susceptible allele.

**SNP ID**	**Significance from previous study**	**Reference**	**Alt allele frequency in Indian database**
rs10900585 (G>T)	G- associated with decreased risk of SM. (African population) **(SMPA)**<br>G- reduced malaria anaemia, protect against malaria in pregnancy. (African population)	19<br><br>27	0.8832
rs10751450 (C>T)<br>rs10751451 (C>T)<br>rs10751452 (T>C)	Associated with PMCA expression, RBC traits, malaria susceptibility (multicentre study, African population)<br>Risk genotype for severe malaria and mild malaria in Sangelese population - CC, CC, TT (African population) **(SMSA)**	21<br>22<br>63<br>61<br>62	0.8857<br>0.8803<br>0.8802
rs1541252 (T>C)<br>rs1541253 (T>C)<br>rs1541254 (C>G)	Risk genotype for severe malaria and mild malaria - CC, CC, GG (African population) **(SMSA, MMSA)**<br>TT, TT, CC- low PMCA in healthy volunteers in Hungary. (European population)<br>TT-Reduced PMCA expression, slow growth of Pf. (Gambia)	20<br>23<br>61<br>62	0.8802<br>0.8743<br>0.8834
rs15411255 (G>A)	GG- reduced parasite density.	24	0.8743
	PMCA inhibitor reduced malaria parasite growth.	52	

Table 2: Hardy Weinberg equilibrium analysis of 14 ATP2B4 SNPs.

SNPs	chi-square test statics	P-value of chi-square
rs11240733 (T>C)	0.00012013	0.991255051
rs10751449 (A>T)	0.00729955	0.931913657
rs10736845 (C>T)	0.00729955	0.931913657
rs10751450 (T>C)	0.051870221	0.81984033
rs10751451 (T>C)	7.12142E-08	0.999787077
rs10751452 (C>T)	0.003703729	0.951472087
rs1541252 (C>T)	0.0013571	0.970613504
rs1541253 (C>T)	0.0013571	0.970613504
rs1541254 (G>C)	0.004161543	0.94856413
rs1541255 (A>G)	0.004161543	0.94856413
rs1419114 (G>A)	0.00068943	0.979052353
rs10900585 (T>G)	0.175234726	0.675500836
rs10900586 (A>G)	0.015020412	0.90245728
rs10793762 (C>T)	0.01115214	0.915896793

Table 3. Prevalence of genotypes in Different Sample Groups. This table displays the prevalence of major, minor, and heterozygous genotypes present in three different regulatory regions of the gene in different malaria outcome, including malaria positive, malaria negative, febrile but malaria negative and severe malaria of Indian population.

SNPs	Genotypes	Malaria negative Healthy N=117	Malaria negative Febrile N=40	Malaria Positive N= 54	Severe malaria N=24	*p*-value of chi-square test
**rs11240733 rs10751449 rs10736845 rs10751450 rs10751451 rs10751452**	Major Genotype (SMPA/MMPA) **TACTTC/TACTTC**	**79**	**35**	**45**	**20**	**0.0559**
	Hetero **TACTTC/CTTCTT**	**21**	**1**	**3**	**2**	
	Minor Genotype **CTTCCT/CTTCTT**	**17**	**4**	**6**	**2**	

**rs1541252<br>rs1541253<br>rs1541254<br>rs1541255<br>rs1419114**	Major Genotype<br>(SMSA/MMSA)<br>**CCGAG/CCGAG**	**89**	**35**	**49**	**20**	**0.0975**
	Hetero<br>**CCGAG/TTCGA**	**24**	**3**	**2**	**3**	
	Minor Genotype<br>**TTCGA/TTCGA**	**4**	**2**	**3**	**1**	
**rs10900585<br>rs10900586<br>rs10793762**	Major Genotype<br>(SMSA/MMSA)<br>**TAC/TAC**	**84**	**33**	**49**	**19**	**0.1280**
	Hetero<br>**TAC/ GGT**	**25**	**5**	**2**	**4**	
	Minor Genotype<br>**GGT/GGT**	**8**	**2**	**3**	**1**	

Table 4: PCR condition and primers used for the amplification of regulatory DNA region of ATP2B4.

**Gene/ region**	**Primers**	**Amplicon size**	**1° PCR**	**2° PCR**
ATP2B4 (Enhancer)	5’AGAGGATTGTCAGGAATCGGC3’<br>5’TCCAGGTGTATGGAAATGACAAC3’	887bp	95°C-3min<br>95°C-30sec<br>55°C-1 min<br>72°C-4 min<br>72°c-10min	95°C-5min<br>95°C-30 sec<br>63°C-30sec<br>72°C-1 min<br>72°C-5 min
ATP2B4 (5’UTR)	5’ACTGGGTACCTCTTGCCCTT3’<br>5’CACTAGCCACCTTCCTCCAA3’	749bp		
ATP2B4 (2nd intron)	5’TGGGATGCTAAATCACAAGGT3’<br>5’GGCCAAGTAAGAATCTGCTACA3’	603bp		

Table 5: Samples used in the study.

1. Severe malaria

Sl.No.	Age	Sex	Fever	Headache	Vomiting	Myalgia	Convulsion	Unconscious-ness	Decreased Urination	Black Urine	Diarrhea	Jaundice	Hepatomegaly	Spleenomegaly	Bleeding	QBC	ICT	Parasitemia(/µL)	Temp (°F)	Hb (gm/dl)	GCS	N	L	E	B	M	TLC ($10^3/mm^3$)	RBS (gm/dl)	TPC ($10^3/mm^3$)	Urine output ml/day	B.Urea (mg/dl)	S.Cr (mg/dl)	CRP(mg/L)	S.Bil (mg/dl)	SGOT (AST) (IU/L)	SGPT (ALT) (IU/L)	ALP(IU/L)	Organ dysfunction
S1	24	F	+	+	+	+	+	+	+	-	-	+	+	+	-	++	+	7536	101	11	7	82	12	6	0	0	5.5	54	135	400	112	4.4	84	3.2	58	62	88	CM + ARF + Jaundice
S2	32	M	+	+	+	-	+	+	-	-	-	-	-	-	-	+++	+	9822	101.2	7.6	8	76	20	4	0	0	4.56	76	124	1500	34	1.1	124	1.4	74	66	100	CM
S3	44	M	+	-	+	-	-	+	+	-	-	-	-	-	-	++		8456	102	9.6	6	88	9	3	0	0	6.45	70	145	600	86	3.2	156	1.2	66	48	88	CM + ARF + ANAEMIA
S4	26	M	+	+	+	-	-	-	+	-	-	+	+	+	-	+		6720	100	9	14	70	27	2	1	0	6.6	102	210	500	128	4.6	112	6.4	72	66	102	ARF + ANAEMIA + Jaundice
S5	70	M	+	+	-	+	-	+	+	-	-	+	+	+	-	+++		7890	103	10	8	75	25	0	0	0	8.6	55	220	200	312	6.8	98	7.6	57	58	78	CM +ARF + Jaundice
S6	45	F	-	+	+	+	+	+	+	-	-	+	+	-	-	+		8544	98.6	7.6	7	80	17	3	0	0	9.2	62	126	600	210	7.2	146	2.8	63	66	84	CM +ARF + Jaundice +ANAEMIA
S7	24	M	+	+	+	+	-	-	+	+	-	+	+	+	+	++++		6452	102.4	6.5	15	81	11	8	0	0	10.4	100	54	350	180	3.4	168	3.8	77	68	94	ARF + Jaundice + ANAEMIA + Thrombocytopenia
S8	55	M	+	+	-	+	-	-	+	-	-	-	-	-	-	+++		7328	101.2	10.6	15	77	19	4	0	0	6.5	146	164	300	98	2.4	88	0.8	46	40	110	ARF
S9	25	F	-	+	+	+	+	+	-	+	-	+	+	+	-	+	+	9436	99.8	8.6	8	76	23	0	1	0	6.4	80	208	1600	24	1.1	46	4.2	54	58	78	CM + ANAEMIA + Jaundice
S10	44	M	+	+	-	-	-	-	+	-	-	-	-	-	-	++		9348	100.4	10	15	68	30	1	1	0	4.6	124	166	400	244	6.4	142	0.8	44	36	122	ARF
S11	55	M	+	-	+	+	-	+	+	-	-	+	+	-	-	+++		6852	101.4	9.8	7	88	8	4	0	0	9.8	58	124	500	124	3.4		8.4	56	67	101	CM + ARF +Jaundice +ANAEMIA
S12	33	F	+	+	+	-	-	-	+	-	+	+	+	+	-	++++		7988	102	10	14	80	18	2	0	0	10.4	120	314	400	344	8.4		2.8	65	73	79	ARF+ Jaundice
S13	40	F	+	+	+	+	+	+	-	-	-	-	-	-	-	++	+	6456	102	6	7	70	28	1	1	0	11.4	76	150	2000	26	0.9		1.4	45	34	86	CM
S14	56	F	+	-	+	-	-	-	+	-	-	+	+	+	-	++	+	7844	104	7	15	72	27	0	1	0	10.4	110	315	600	128	3.6	142	12.2	78	66	104	ARF+ Jaundice+ANAEMIA
S15	20	F	+	+	-	-	+	+	-	-	-	-	+	+	-	+++		8456	103	10.6	8	78	22	0	0	0	9	142	156	1400	30	0.8		1.2	35	44	124	CM
S16	42	M	+	+	+	-	+	+	-	-	-	-	-	+	-	++		9434	102.4	8	10	87	10	3	0	0	8.6	90	162	1200	34	1		0.8	55	40	78	CM
S17	22	M	-	+	+	+	+	+	+	-	-	+	+	-	-	+		6788	101.2	9.2	8	66	29	4	0	1	5.86	46	315	200	280	6.2	164	10.2	82	77	98	CM+ARF+Jaundice +ANAEMIA
S18	25	M	+	+	+	+	+	-	+	-	-	-	-	+	-	++	+	8244	99.8	11	15	77	23	0	0	0	9.4	156	214	100	312	7	187	1.3	34	32	76	ARF
S19	45	F	+	+	+	+	-	-	+	+	-	+	+	+	+	+++		8466	100	6	15	83	17	0	0	0	5.8	124	60	400	110	5.4	124	4.8	56	77	88	ARF+Jaundice+ANAEMIA+Thrombocytopenia
S20	20	F	+	+	+	+	-	-	+	-	-	-	-	-	-	++		9856	101.2	10.4	14	80	20	0	0	0	9.6	110	246	550	96	3.8		1.2	54	46	120	ARF
S21	60	M	+	-	-	+	+	+	-	-	-	-	+	+	-	++++		8344	101	11.2	15	76	22	1	0	1	10.4	100	214	2000	38	1.1		6	88	79	102	CM

GCS Glasgow Coma Scale score ≤ 9, SOD- single organ dysfunction; MODS-multi organ dysfunction; CM-cerebral malaria; SMA-severe malaria anemia; ARF-acute renal failure.

2. Uncomplicated *Plasmodium falciparum* malaria

SL NO	SITE	VILLAGE	Endemicity	CODE NO	AGE GROUP	SEX	*P.f.* (RDT)
1	BIRSHA	KUNDEKASHA	High	BK - 1	15 & ABOVE	M	positive
2	BIRSHA	KUNDEKASHA	High	BK - 2	15 & ABOVE	M	positive
3	BIRSHA	KUNDEKASHA	High	BK - 3	15 & ABOVE	F	positive
4	BIRSHA	KUNDEKASHA	High	BK - 4	15 & ABOVE	M	positive
5	BIRSHA	KUNDEKASHA	High	BK - 5	15 & ABOVE	M	positive
6	BIRSHA	KUNDEKASHA	High	BK - 6	15 & ABOVE	M	positive
7	BIRSHA	KUNDEKASHA	High	BK - 7	15 & ABOVE	F	positive
8	BIRSHA	KUNDEKASHA	High	BK - 8	15 & ABOVE	F	positive
9	BIRSHA	KUNDEKASHA	High	BK - 9	15 & ABOVE	F	positive
10	BIRSHA	KUNDEKASHA	High	BK - 10	15 & ABOVE	M	positive

11	BIRSHA	KUNDEKASHA	High	BK - 11	15 & ABOVE	F	positive
12	BIRSHA	KUNDEKASHA	High	BK - 12	15 & ABOVE	F	positive
13	BIRSHA	KUNDEKASHA	High	BK - 13	15 & ABOVE	F	positive
14	BIRSHA	KUNDEKASHA	High	BK - 14	15 & ABOVE	M	positive
15	BIRSHA	KUNDEKASHA	High	BK - 15	15 & ABOVE	F	positive
16	BIRSHA	KUNDEKASHA	High	BK - 16	15 & ABOVE	M	positive
17	BIRSHA	KUNDEKASHA	High	BK - 17	15 & ABOVE	M	positive
18	BIRSHA	KUNDEKASHA	High	BK - 18	15 & ABOVE	F	positive
19	BIRSHA	KUNDEKASHA	High	BK - 19	15 & ABOVE	M	positive
20	BIRSHA	KUNDEKASHA	High	BK - 20	15 & ABOVE	M	positive
21	BIRSHA	KUNDEKASHA	High	BK - 21	15 & ABOVE	F	positive
22	BIRSHA	KUNDEKASHA	High	BK - 22	15 & ABOVE	F	positive
23	BIRSHA	KUNDEKASHA	High	BK - 23	15 & ABOVE	M	positive
24	BIRSHA	KUNDEKASHA	High	BK - 24	15 & ABOVE	M	positive
25	BIRSHA	KUNDEKASHA	High	BK - 25	15 & ABOVE	F	positive
26	BIRSHA	KUNDEKASHA	High	BK - 26	15 & ABOVE	F	positive
27	BIRSHA	KUNDEKASHA	High	BK - 27	15 & ABOVE	F	positive
28	BIRSHA	KUNDEKASHA	High	BK - 28	15 & ABOVE	M	positive
29	BIRSHA	KUNDEKASHA	High	BK - 29	15 & ABOVE	F	positive
30	BIRSHA	KUNDEKASHA	High	BK - 30	15 & ABOVE	F	positive
31	BIRSHA	KUNDEKASHA	High	BK - 31	15 & ABOVE	F	positive
32	BIRSHA	BONDARI	High	BB - 1	15 & ABOVE	M	positive
33	BIRSHA	BONDARI	High	BB - 2	15 & ABOVE	F	positive
34	BIRSHA	BONDARI	High	BB - 3	15 & ABOVE	M	positive
35	BIRSHA	BONDARI	High	BB - 4	15 & ABOVE	M	positive

36	BIRSHA	BONDARI	High	BB - 5	15 & ABOVE	F	positive
37	BIRSHA	BONDARI	High	BB - 6	15 & ABOVE	F	positive
38	BIRSHA	BONDARI	High	BB - 7	15 & ABOVE	M	positive
39	BIRSHA	BONDARI	High	BB - 8	15 & ABOVE	M	positive
40	BIRSHA	BONDARI	High	BB - 9	15 & ABOVE	M	positive
41	BIRSHA	BONDARI	High	BB - 10	15 & ABOVE	M	positive
42	BIRSHA	BONDARI	High	BB - 11	15 & ABOVE	M	positive
43	BIRSHA	BONDARI	High	BB - 12	15 & ABOVE	M	positive
44	BIRSHA	BONDARI	High	BB - 13	15 & ABOVE	M	positive
45	BIRSHA	BONDARI	High	BB - 14	15 & ABOVE	M	positive
46	BIRSHA	BONDARI	High	BB - 15	15 & ABOVE	M	positive
47	BIRSHA	BONDARI	High	BB - 16	15 & ABOVE	F	positive
48	BIRSHA	BONDARI	High	BB - 17	15 & ABOVE	F	positive
49	BIRSHA	BONDARI	High	BB - 18	15 & ABOVE	M	positive
50	BIRSHA	BONDARI	High	BB - 19	15 & ABOVE	F	positive
51	BIRSHA	BONDARI	High	BB - 20	15 & ABOVE	F	positive
52	BIRSHA	BONDARI	High	BB - 21	15 & ABOVE	F	positive
53	BIRSHA	CHIKHLI	High	BC - 1	15 & ABOVE	M	positive
54	BIRSHA	CHIKHLI	High	BC - 2	15 & ABOVE	F	positive
55	BIRSHA	CHIKHLI	High	BC - 3	15 & ABOVE	F	positive
56	BIRSHA	CHIKHLI	High	BC - 4	15 & ABOVE	M	positive
57	BIRSHA	CHIKHLI	High	BC - 5	15 & ABOVE	F	positive
58	BIRSHA	CHIKHLI	High	BC - 6	15 & ABOVE	F	positive
59	BIRSHA	CHIKHLI	High	BC - 7	15 & ABOVE	F	positive
60	BIRSHA	CHIKHLI	High	BC - 8	15 & ABOVE	F	positive

3. Samples for in-vitro studies

S.N	*P.f.* (RDT)	*P.v.* (RDT)	RBC	HGB	HCT	MCV	MCH	MCHC	G6PD Deficiency	HbA/S/C
			3.80-5.80*10^6/microL	11.5-17.5 g/dl	35-50 %	82-100 fL	27-34 pg	31.6-35-4 g/Dl		
S1	Negative	Negative	4.43	15.7	46.7	105.5		33.7	No	AA
S2	Negative	Negative	347	12.9	37.9	109.1		34.1	No	AA
S3	Negative	Negative	4.12	13	37.9	92.2		34.4	No	AA
S4	Negative	Negative	6.65	13.6	41.8	62.7	20.5	32.6	No	AA
S5	Negative	Negative	4.43	14.3	42	94.8	32.2	34	No	AA
S6	Negative	Negative	3.88	10	31.6	81.3		31.8	No	AA
S7	Negative	Negative	4.2	10.5	33.2	79	25	31.7	No	AA
S8	Negative	Negative	4.51	12.6	38.5	85.3	27.8	32.6	No	AA
S9	Negative	Negative	4.5	11.4	35.6	79	25.2	32	No	AA
S10	Negative	Negative	4.49	13	40.6	90.5	29	32	No	AA
S11	Negative	Negative	4.74	14.4	43.5	91.7	30.4	33.1	No	AA
S12	Negative	Negative	5.78	17.4	51.5	89.1	30.1	33.8	No	AA
S13	Negative	Negative	3.21	6.4	21	65.4	19.8	30.2	No	AA
S14	Negative	Negative	4.09	9.6	30.4	74.4	23.5	31.6	No	AA
S15	Negative	Negative	5.22	15.4	46.4	88.8	29.6	33.3	No	AA
S16	Negative	Negative	4.7	14.9	44.8	95.3	31.6	33.2	No	AA
S17	Negative	Negative	5.5	12.2	30.4	67.7	22.2	32.7	No	AA
S18	Negative	Negative	5.61	11.7	37.6	67		31	No	AA
S19	Negative	Negative	3.58	11.7	34.3	95.7	32.7	34.2	No	AA
S20	Negative	Negative	4.2	12.2	37.3	88.7	29	32.7	No	AA
S21	Negative	Negative	4.62	14.2	43.1	93.2	30.8	33	No	AA
S22	Negative	Negative	4.47	11.4	35.5	79.5		32.2	No	AA
S23	Negative	Negative	4.72	11	41.3	87.5		33.9	No	AA
S24	Negative	Negative	4.82	14	42.5	88.3	29.1	32.9	No	AA
S25	Negative	Negative	4.17	12.5	37.7	90.4	30	33.2	No	AA
S26	Negative	Negative	4.33	12.5	36.7	84.8	28.9	34.1	No	AA
S27	Negative	Negative	4.93	14.8	45	91.3	30	32.9	No	AA
S28	Negative	Negative	5.35	16.6	50	93.5	30.9	33.1	No	AA
S29	Negative	Negative	5.21	15.3	45.1	86.6	29.3	33.9	No	AA
S30	Negative	Negative	4.59	13.1	40.5	88.3	28.6	32.4	No	AA
S31	Negative	Negative	3.61	11.5	37.5	85.4	29.2	32.7	No	AA
S32	Negative	Negative	4.3	12.1	42.5	96.2	33.7	31	No	AA

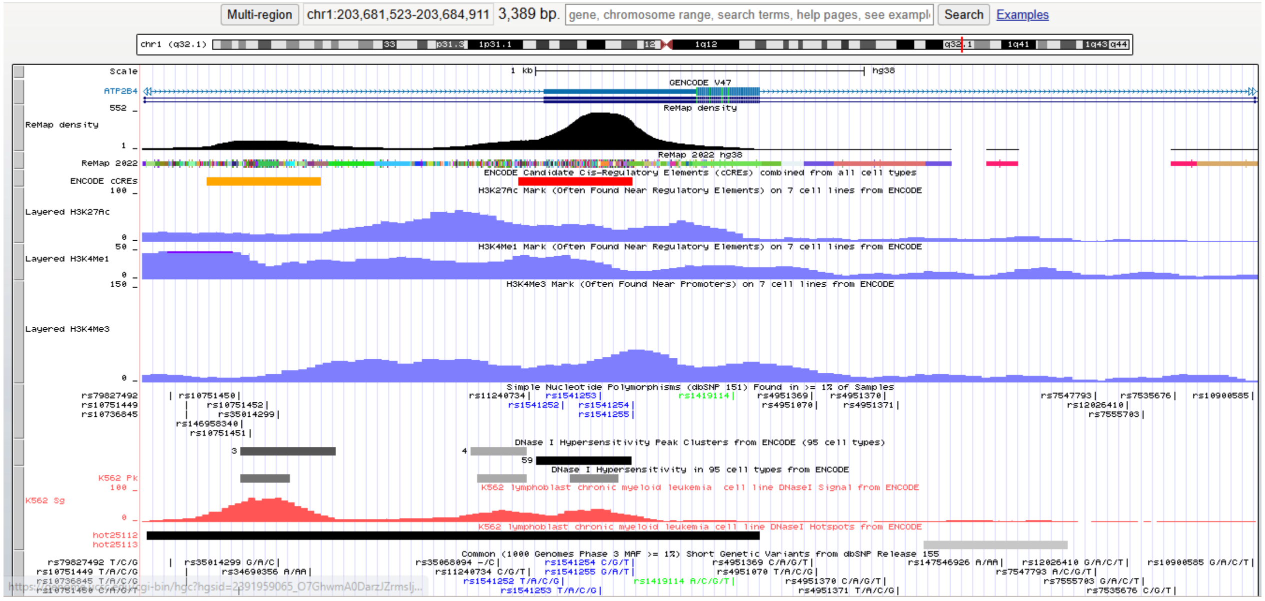

Figure 1: ATP2B4 regulatory regions identified by UCSC genome browser. ATP2B4 region ch1:203681464-203684970 containing the 8 variants studied for regulatory mechanism. **[A]** Remap tool is a computational platform used for analysing and interpreting functional genomics data. It integrates various publicly available ChIP-seq (Chromatin Immunoprecipitation sequencing) datasets to identify regulatory elements such as enhancers, promoters, and transcription factor binding sites across the human genome. Out of 3 regions, enhancer and 5'UTR region are located within main peaks of Remap density. Candidate Cis-Regulatory Elements [cCREs] in the human genome are displayed. Red colour indicates cCREs with promoter-like signatures [cCRE-PLS] in 5'UTR region and orange colour indicate proximal cCREs enhancer-like signatures [cCRE-ELS] in enhancer region. H3K27Ac (acetylation of lysine 27 of the H3 histone protein) and H3K4me1 (tri-methylation of lysine 4 of the H3 histone protein) marks are associated with enhancers showing peaks in enhancer and 5'UTR, while H3K4me3 is associated with active promoters showing main peak in 5'UTR. DNase I hypersensitivity regions display peaks at enhancer and 5'UTR sites, indicating their regulatory role in ATP2B4 gene transcription.

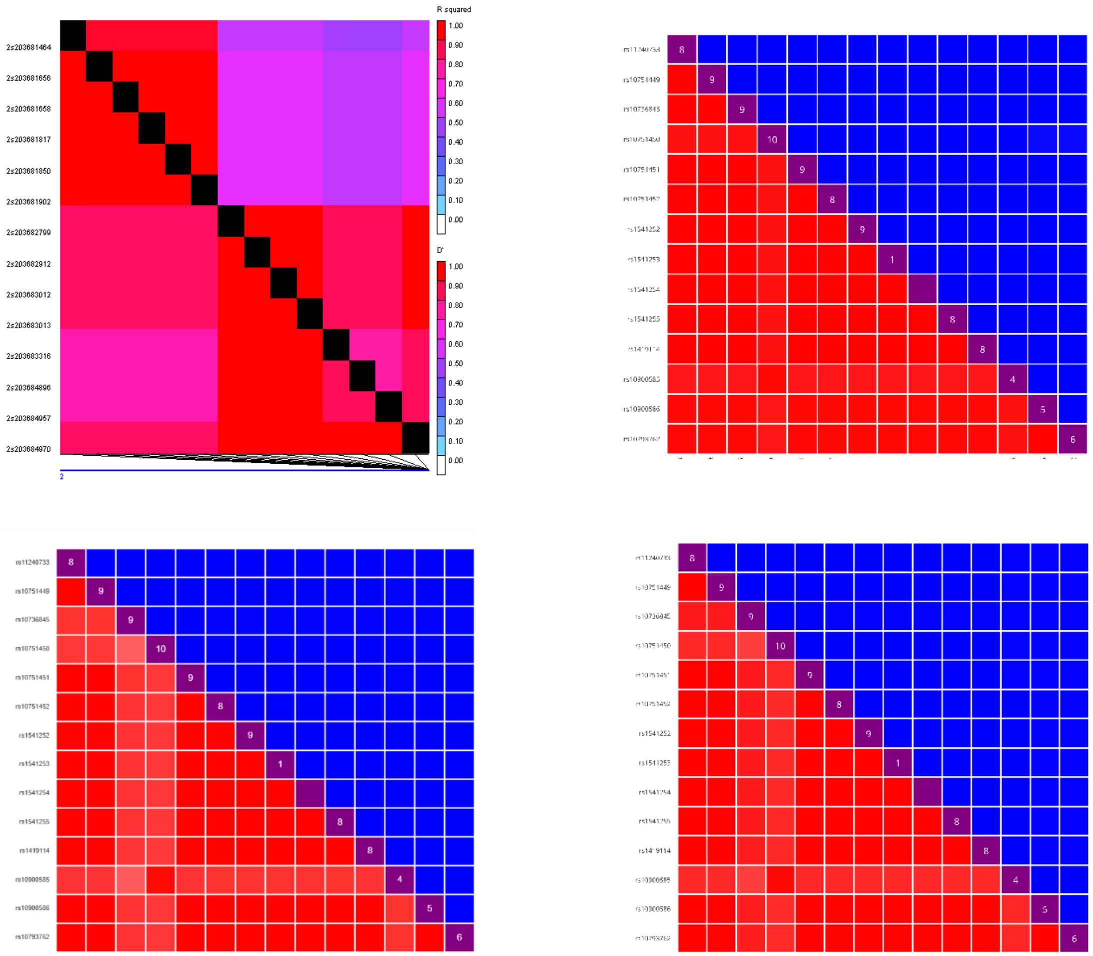

Figure 2: Linkage Disequilibrium plot of 14 SNPs of ATP2B4 across populations of 1000 genome project. The LD (Linkage Disequilibrium) plot showcases the patterns of pairwise linkage disequilibrium, measured by R-squared (r^2) and D' statistics, among 14 SNPs situated in the regulatory regions of ATP2B4 gene variants within (A) studied Indian population (B) African population (1000genome) and (C) Southeast Asian population (1000genome) and (D) all population of 1000genome project. The plot employs color-coded representation to signify the strength of LD between SNP pairs.

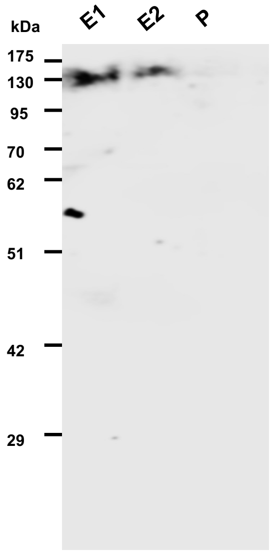

Figure 3. Validation of PMCA4b antibody specificity in RBCs. Immunoblot probed with anti-PMCA4b monoclonal antibody (clone JA3, Merck Millipore) and HRP-conjugated goat anti-mouse secondary antibody. A distinct band was detected at ~130 kDa in RBC ghosts (E1 and E2), consistent with the expected molecular weight of PMCA4b, whereas no signal was observed in saponised parasite lysate (P).

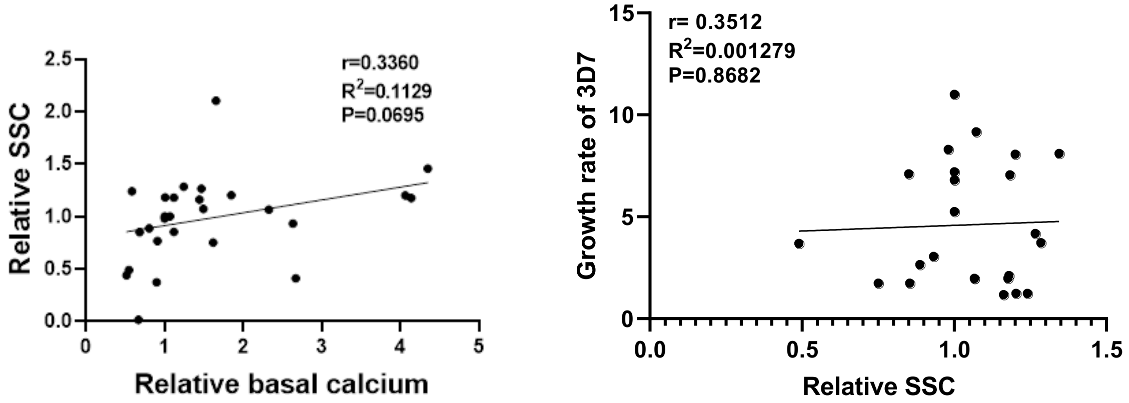

Figure 4: Relationship between calcium level and SSC. (A) Scatter plot depicting the relationship between basal calcium levels and relative side scatter of RBCs. (B) Scatter plot depicting the relationship between *P. falciparum* growth rate and relative side scatter of RBCs.

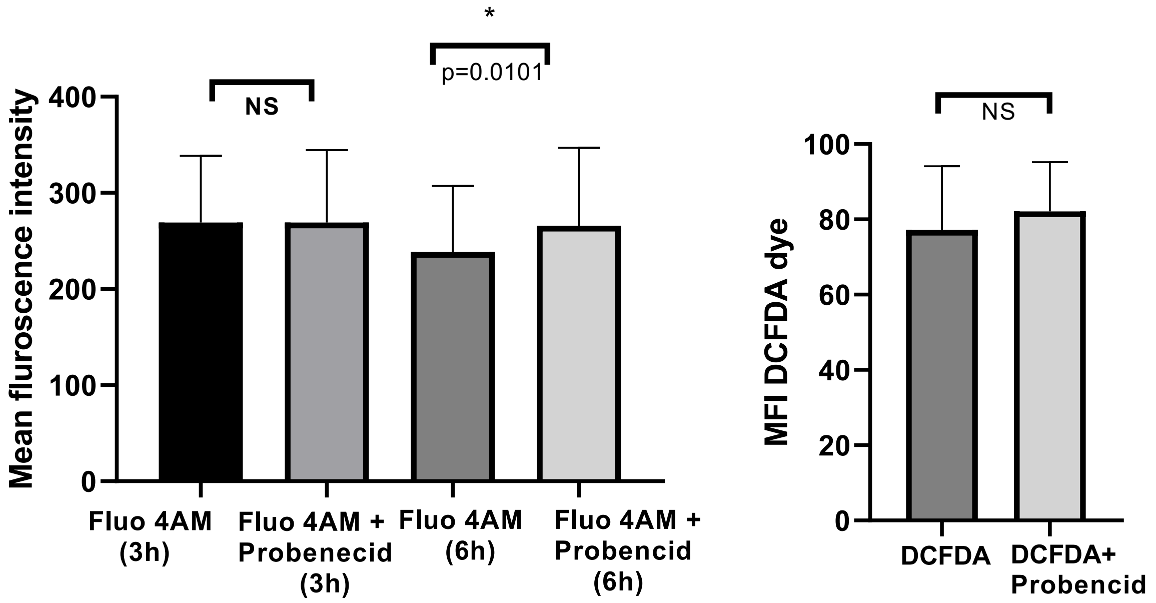

A B

Figure 5. Effect of probenecid (2.7 mM) on dye retention in RBCs after dye loading. Quantitative comparison of mean fluorescence intensity (MFI) values from six independent experiments (n = 6) showing no significant effect of probenecid on intracellular DCFDA or Fluo 4AM dye levels 3 hours post-loading. Probenecid shows significant effect on dye retention after 6-hour post loading (P = 0.0101, t = 4.027, df = 5).

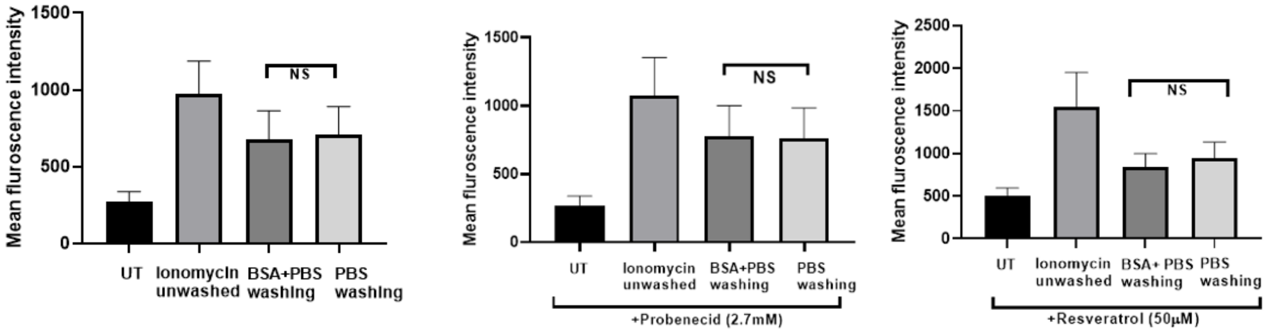

A B C

Figure 6. Effect of washing conditions (PBS vs. BSA) on Fluo 4AM loaded RBCs under different treatments. (A) Comparison of Fluo-4 fluorescence in RBCs after ionomycin stimulation without probenecid shows no significant difference between PBS and BSA washed samples. (B) In parallel, when RBCs were incubated with Fluo-4 in the presence of probenecid, the subsequent comparison of PBS and BSA washes again revealed no significant difference. (C) Treatment of washed RBCs with resveratrol (50 µM, 2 h) also did not result in any significant difference in Fluo-4 signal between PBS and BSA washes.

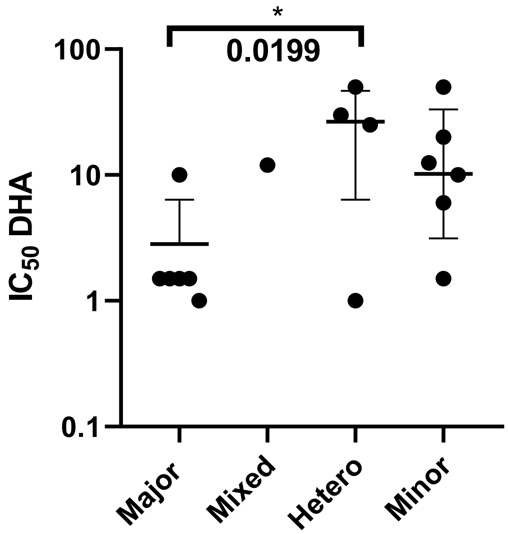

Figure 7: Relationship between ATP2B4 genotypes and artemisinin sensitivity. The IC50 (nM) values obtained for each sample were plotted with their respective genotype. *p*-values are indicated in the figure.
